# Immunomodulatory Roles and Clinical Significance of GZMM and DDX24 in Sepsis: A Multiomics Integrative Analysis With Experimental Validation

**DOI:** 10.1155/humu/4951633

**Published:** 2026-04-03

**Authors:** Yi Zhang, Liang Tang, Juan Wu, Lin Yang, Wen Liu, Yi Liang, Jianfang Han, Shuang He, Yulian Yang

**Affiliations:** ^1^ Department of Critical Care Medicine, The Second People′s Hospital of Deyang, Deyang, Sichuan Province, China; ^2^ Department of Critical Care Medicine, The Affiliated Hospital, Southwest Medical University, Luzhou, Sichuan Province, China, swmu.edu.cn

**Keywords:** bioinformatics, biomarker, DDX24, GZMM, immune regulation, scRNA-seq, sepsis

## Abstract

Sepsis is a systemic inflammatory response syndrome caused by an infection featuring high morbidity and mortality due to complex mechanisms underlying immune dysfunction. In this study, based on the sepsis transcriptome profiles from the GEO datasets (GSE65682, GSE28750, GSE95233, and GSE167363), we used the machine learning method and other computational algorithms, such as differential gene expression analysis, weighted gene coexpression network analyses (WGCNA), and the building of PPI networks to identify four hub genes (DDX24, GZMM, KCNA3, and NCL). The quantitative reverse transcription PCR performed preliminary validation that all four hub genes were significantly downregulated in patients with sepsis. DDX24 had the highest diagnostic performance (AUC > 0.8) for discriminating patients from normal subjects. GZMM was found to be significantly related to the prognoses of patients as well as APACHE II scores, and the downregulated expression pattern might represent T cell and NK cell exhaustion. Analysis based on single‐cell RNA sequencing showed that DDX24 and GZMM were mainly expressed in T cells and NK cells, and the expression trends strongly correlate with patient survival. Functional enrichment analysis suggested that the hub genes likely participate in regulation of immune responses, especially those pertaining to T cells. Drug prediction found 25 candidate drugs that will serve as new therapeutic targets for precision medicine to treat sepsis. Overall, the multifaceted study shed light on key roles played by these hub genes (especially DDX24 and GZMM) in the development of sepsis and will be useful references in diagnosing patients and estimating prognosis.

## 1. Introduction

Sepsis is an ongoing health problem associated with a high mortality rate among intensive care unit (ICU) patients around the world [1]. The World Health Organization statistics estimate that sepsis affects nearly 30 million people each year and approximately half of these cases occur in low‐ and middle‐income countries [2].

Pathogens associated with sepsis are broad and include the common gram‐negative bacteria *Escherichia coli* [3], gram‐positive bacteria *Staphylococcus aureus* [4], and fungi *Candida* [5] and *Aspergillus* [6]. Infections from these organisms may result in SIRS after causing respiratory [7], GI/abdominal [8], skin/soft tissue [9], and urinary tract [10] infections among others. Viral sepsis can occur following severe flu infection [11]. Due to such pathogenic diversity, studies focusing on host immune mechanisms and biomarker identification have gained significance for research.

In recent developments on biomarker research, many biomarkers used in sepsis diagnosis and prognosis have been proposed. Research found that HGB and ALB level [12, 13], and PCT/ALB, PCT/CHOL, and NEU/CHOL [14, 15] ratios were very useful in diagnosing and forecasting prognosis of elderly sepsis patients [16]. Machine learning techniques can significantly enhance prediction performance to detect community‐acquired pneumonia patients for intensive care support [17, 18].

Gene expression profiling is a powerful tool for investigating sepsis molecular mechanisms. Comparative analyses between healthy controls and sepsis patients have identified disease‐associated candidate genes. For instance, SGK1, DYSF, and MSRB1 demonstrate excellent diagnostic performance for sepsis‐induced acute respiratory distress syndrome [19]. Significant alterations in vitamin D and its metabolites in sepsis patients, closely associated with gut microbiota composition, have also been reported [20]. Additionally, certain long noncoding RNAs regulate immune responses during sepsis progression [21]. These findings enhance our understanding of sepsis pathophysiology and provide potential targets for novel diagnostics.

In this study, we performed comprehensive bioinformatics analyses using sepsis‐related transcriptome data from the GEO database, coupled with qRT‐PCR validation in clinical peripheral blood samples. Through integrated bulk transcriptomic and single‐cell level investigations, we systematically identified key genes associated with sepsis pathogenesis and patient outcomes, providing theoretical support for clinical diagnosis and treatment.

## 2. Materials and Methods

### 2.1. Public Data Sources

Sepsis‐related transcriptomic datasets were downloaded from the GEO database (https://www.ncbi.nlm.nih.gov/geo/). The GSE65682 dataset (802 whole‐blood samples: 760 sepsis, 42 healthy controls; and 479 with survival information) served as the training set for hub gene screening, prognostic evaluation, and functional analysis. The GSE28750 (41 whole‐blood samples: 21 sepsis vs. 20 controls) and GSE95233 (124 whole‐blood samples: 102 sepsis vs. 22 controls) datasets were validation sets for diagnostic performance assessment. The GSE167363 dataset (12 whole‐blood samples: 2 sepsis deaths at 0 h, 3 sepsis survivors at 0 h, 2 sepsis deaths at 6 h, 3 sepsis survivors at 6 h, and 2 healthy controls) was used for single‐cell RNA sequencing (scRNA‐seq) analysis.

### 2.2. Differentially Expressed Gene (DEG) Screening

The limma R package (v3.5) identified DEGs in the training set (adjusted *p* < 0.05, |logFC| > 1). Volcano plots and heatmaps were generated using ggVolcano (v0.0.2) and ComplexHeatmap (v2.22.0), respectively.

Functional enrichment analysis of DEGs: Gene Ontology (GO) and Kyoto Encyclopedia of Genes, Genomes (KEGG), Reactome, and WikiPathway enrichment analyses was performed using ClusterProfiler R package (v4.14.4) with parameters: p value cutoff = 0.05, P − adjustment method =  ^“^BH^”^, q − value cutoff = 0.05, minimum gene set size = 5.

### 2.3. Weighted Gene Coexpression Network Analysis (WGCNA)

Using the WGCNA R package (v1.73), we constructed a coexpression network based on the training set, with disease status as the primary trait. Key steps are included as follows: (1) Data preprocessing: Selecting the Top 5000 most variable genes and removing outlier samples to establish a normalized expression matrix. (2) Network construction: Determining the optimal soft‐thresholding power (*β* = 13) via topological overlap matrix (TOM) analysis, which was selected as the smallest power value satisfying the scale‐free topology fit index (*R* ^2^) approaching 0.9 to ensure scale‐free network properties. (3) Module identification: Applying dynamic tree cutting and module merging algorithms to hierarchically cluster genes, yielding distinct functional modules characterized by module eigengenes (MEs).

### 2.4. Protein–Protein Interaction (PPI) Network Analysis

The STRING database (https://cn.string-db.org/) constructed a PPI network for DEGs (confidence threshold = 0.7). Hub genes were identified using Cytoscape (v3.9.1) with MCODE (degree cutoff = 2, node score cutoff = 0.2, k − core = 2) and CytoHubba (default parameters) plugins.

### 2.5. Hub Gene Prioritization

Two machine learning approaches ranked hub gene importance: (1) Random forest (Boruta algorithm, v8.0.0, significance threshold *p* = 0.001). 2) Support Vector Machine‐Recursive Feature Elimination (SVM‐RFE, e1071 v1.7.16, 10‐fold cross‐validation). Multivariate Cox regression (survminer v0.5.0) and LASSO regression (glmnet v4.1.8, 10‐fold CV) were applied to GSE65682 survival data to identify prognosis‐associated genes.

### 2.6. Clinical Relevance Evaluation of Hub Genes

For survival analysis, patients were stratified into high‐ and low‐expression groups based on median hub gene expression (a widely used standard grouping strategy with potential inherent arbitrariness). Kaplan–Meier (KM) curves were plotted (GraphPad Prism 9), and log‐rank tests were performed (*p* < 0.05 significant). Diagnostic accuracy was assessed via receiver operating characteristic (ROC) curves (MedCalc) using validation sets.

### 2.7. qRT‐PCR Validation

Peripheral blood samples were collected from 35 sepsis patients (27 males, 8 females; Sepsis‐3.0 criteria) and 6 healthy controls (four males, two females) admitted to the ICU of Affiliated Hospital of Southwest Medical University (April–December 2024). Detailed demographic and clinical characteristics (e.g., age, source of infection, SOFA/APACHE II scores, comorbidities, and clinical treatments) of the cohort were not collected in this study. qRT‐PCR quantified expression levels of the four hub genes (DDX24, GZMM, KCNA3, and NCL).

### 2.8. Functional Characterization of Hub Genes

#### 2.8.1. Gene Set Enrichment Analysis (GSEA)

Single‐gene GSEA (GseaVis v0.1.1) was conducted using GO/KEGG terms, ranked by Pearson correlation coefficients between hub genes and all other genes.

#### 2.8.2. Immune Cell Infiltration

The CIBERSORT algorithm estimated the relative abundance of 22 immune cell types. Spearman correlation analysis evaluated associations between hub genes and immune infiltration.

### 2.9. Therapeutic Target Prediction

#### 2.9.1. Transcriptional Regulators

Potential transcription factors (TFs) targeting hub genes were predicted using ChEA3, ENCODE, and JASPAR databases.

#### 2.9.2. miRNA‐mRNA Interactions

Experimentally validated miRNAs were retrieved from miRTarBase v9.0 and TarBase v9.0.

#### 2.9.3. Drug‐Gene Interactions

The DGIdb database identified drugs targeting hub genes and core TFs. Regulatory networks were visualized using Cytoscape.

### 2.10. scRNA‐Seq Analysis

The GSE167363 dataset was processed using Seurat (v5.1.0) with QC thresholds: nFeature_RNA > 100 and < 3000; nCount_RNA > 100 and < 15,000; mitochondrial gene percentage < 20%. Doublets were removed using DoubletFinder (v2.1.3). Data were normalized, the Top 2000 variable genes selected for PCA, and the Top 30 principal components used for *t*‐SNE dimensionality reduction (resolution = 0.6, determined based on cluster stability metrics and biological interpretability of immune cell subsets). Clusters were annotated using CellMarker 2.0 and GO/KEGG enrichment of marker genes. Wilcoxon rank‐sum tests assessed differential hub gene expression across cell populations.

### 2.11. Statistical Analysis

Analyses used GraphPad Prism 9, R (v4.2.1), Cytoscape (v3.8.0), SPSS 26.0, and STRING. Diagnostic performance was evaluated via ROC curves (AUC, sensitivity, specificity, Youden′s index). Normality of all data was first verified using the Kolmogorov–Smirnov test: Parametric unpaired *t*‐tests were applied for group comparisons of normally distributed data, whereas nonparametric Wilcoxon rank‐sum tests were used for nonnormally distributed data (including scRNA‐seq gene expression, immune cell infiltration, and qRT‐PCR validation data). For multiple testing corrections, the Benjamini–Hochberg (BH) method was adopted to control the false discovery rate (FDR) in differential gene expression analysis, functional enrichment analyses (GO/KEGG/Reactome/WikiPathway), and Spearman correlation analyses, with a strict *q*‐value cutoff of 0.05. Statistical tests included the Kolmogorov–Smirnov test for normality; the Unpaired *t*‐test, and the Wilcoxon test for group comparisons. Significance thresholds:  ^∗^ *p* < 0.05,  ^∗∗^ *p* < 0.01,  ^∗∗∗^ *p* < 0.001.

## 3. Results

### 3.1. Differential Gene Expression Analysis

Comparative transcriptomic analysis of 760 sepsis samples versus 42 healthy controls in GSE65682 identified 1058 DEGs (Table S1), comprising 594 downregulated and 464 upregulated genes (Figure 1a,b, Figure S1a,b). GO and KEGG analyses revealed significant enrichment in immune‐related biological processes, including regulation of cytokine production, modulation of T cell activation via MHC Class II protein complex binding, lipopolysaccharide‐binding activity, and G protein‐coupled receptor signaling pathways. DEGs were particularly implicated in T cell differentiation and activation, antigen processing and presentation, cytokine‐cytokine receptor interactions, and the NOD‐like receptor‐signaling pathway (Figure 1c,d). Reactome and WikiPathway analyses consistently demonstrated substantial perturbations in immune‐related pathways (Figure 1c,d), highlighting profound immune dysregulation in sepsis, with emphasis on aberrant T cell‐mediated processes. All DEG screening was corrected for multiple testing using the BH method (*q* < 0.05).

Figure 1Analysis of differentially expressed genes (DEGs) between sepsis patients and healthy controls. (a) Volcano plot of DEGs. Red dots indicate upregulated genes, blue dots indicate downregulated genes, and gray dots represent genes that did not meet the significance threshold. (b) Density heatmap of DEGs. The upper panel shows the density distribution of gene expression across samples (darker brown indicates higher density). The lower panel is a traditional heatmap; the red color key denotes upregulated genes, and the blue denotes downregulated genes. (c) GO enrichment bar plot for DEGs. Bar height represents the number of genes enriched in each GO term. Terms are annotated below as biological process (GO‐BP), cellular component (GO‐CC), and molecular function (GO‐MF). (d) KEGG pathway enrichment bar plot for DEGs. Bar length represents the number of genes enriched in each pathway. Colors represent different KEGG pathway classifications.

**Figure:**
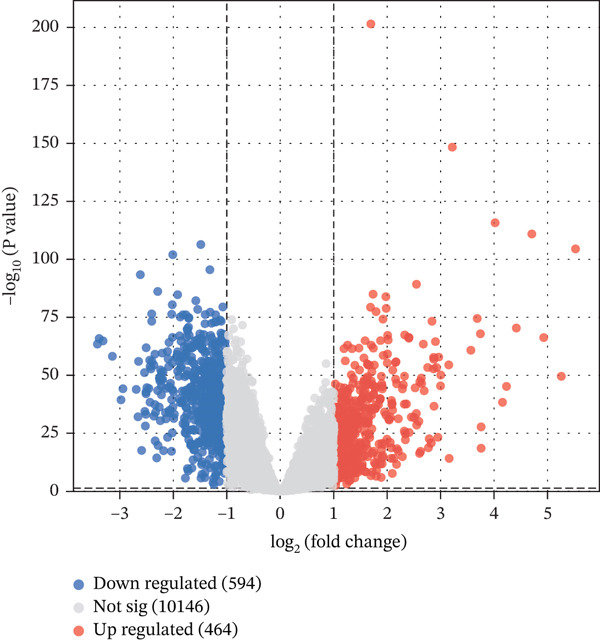
(a)

**Figure:**
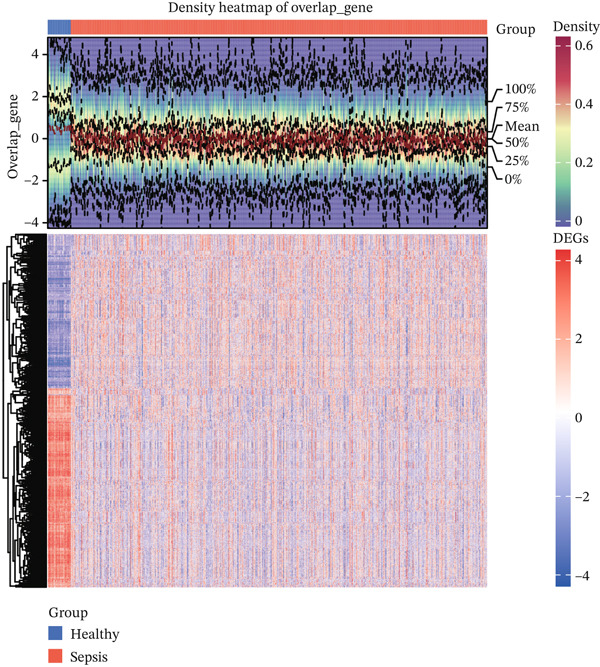
(b)

**Figure:**
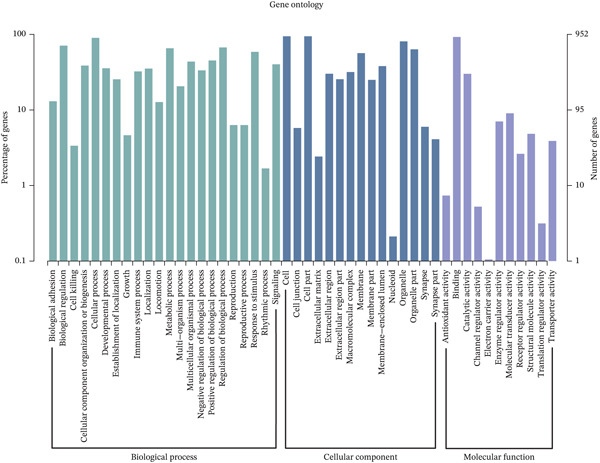
(c)

**Figure:**
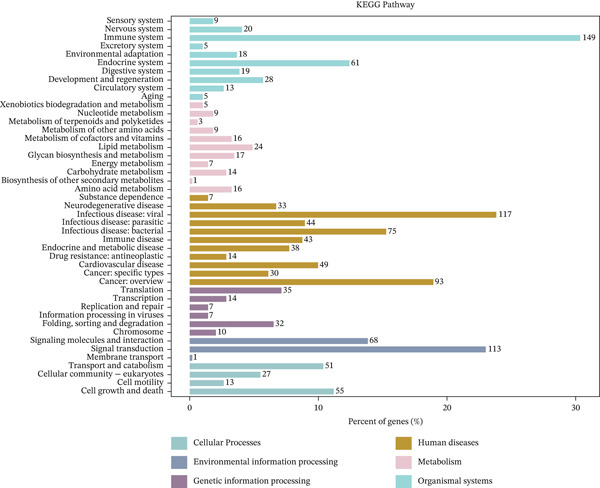
(d)

### 3.2. Identification of Hub Genes

WGCNA on the Top 5000 most variable genes identified coexpression modules. The optimal soft‐thresholding power (*β* = 13) satisfied scale‐free topology criteria (Figure 2a). Hierarchical clustering assigned genes to color‐coded modules (Figure 2b). The yellow module exhibited the strongest correlation with disease status (|*r*| = 0.61, *p* = 2 × 10^−82^), yielding 59 candidate hub genes (Figure 2d, Table S2). PPI network analysis of 1058 DEGs identified 55 core interacting proteins (Figure 2e, Figure 2d). Intersection analysis identified four consensus hub genes: DDX24, KCNA3, NCL, and GZMM (Figure 2f, Table S2). To further elucidate the biological relevance of the yellow module to sepsis pathogenesis, we performed functional enrichment analysis for the 59 genes in this module, and the results showed that these genes were significantly enriched in sepsis‐associated immune and inflammatory biological processes and signaling pathways (adjusted *p* < 0.05). Specifically, the GO enrichment analysis revealed prominent enrichment in T cell activation, regulation of cytokine production, immune response mediated by immunoglobulin, and leukocyte migration, which are the core biological processes underlying immune dysfunction in sepsis. KEGG pathway analysis further demonstrated that the genes in the yellow module were mainly involved in cytokine‐cytokine receptor interaction, NOD‐like receptor‐signaling pathway, and T cell receptor (TCR)‐signaling pathway. All these pathways are well recognized to play key regulatory roles in the occurrence and development of sepsis, including the initiation of inflammatory storm, the imbalance of innate and adaptive immunity, and the exhaustion of T/NK cells. These enrichment results confirm that the yellow module is not only statistically correlated with sepsis status, but also its encoded biological functions are highly consistent with the core pathological mechanisms of sepsis, thus justifying the selection of this module for subsequent hub gene screening.

Figure 2Screening of core genes associated with sepsis. (a) Scale‐free topology fit index (*R* ^2^) and mean connectivity analysis for WGCNA soft‐thresholding power selection. The smallest power value where *R* ^2^ first approaches 0.9 (dashed line) is the recommended optimal soft‐thresholding power. The subsequent plateau near mean connectivity = 0 in the right panel further supports this choice. (b) Gene dendrogram and module assignment. The upper section shows the hierarchical clustering tree (dendrogram) of genes. The colors below assign genes to coexpression modules based on topological overlap. (c) Heatmap depicting module‐trait associations. Red squares indicate a positive correlation, and blue squares indicate a negative correlation. Numbers outside parentheses represent correlation coefficients; numbers inside parentheses indicate *p* values. (d) Protein–protein interaction (PPI) network of core genes. Node color intensity (red) corresponds to the degree of centrality of each gene within the network. (e) Venn diagram illustrating the overlap between genes in the PPI core network and WGCNA modules.

**Figure:**
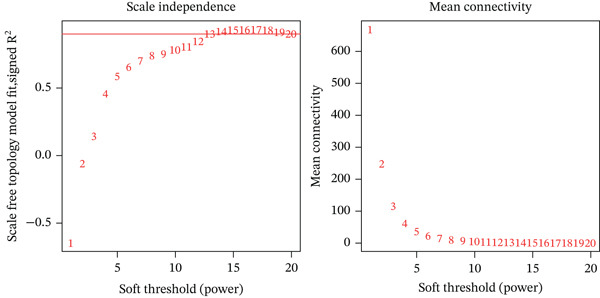
(a)

**Figure:**
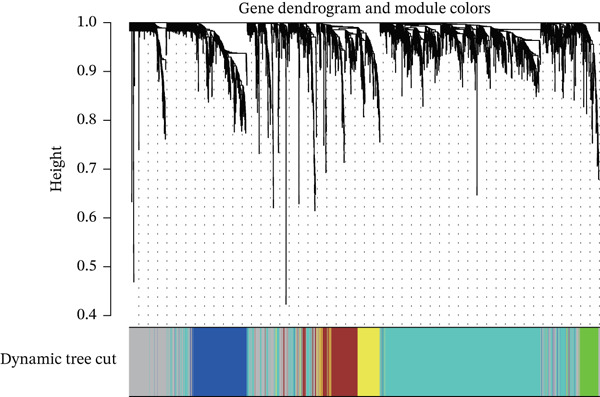
(b)

**Figure:**
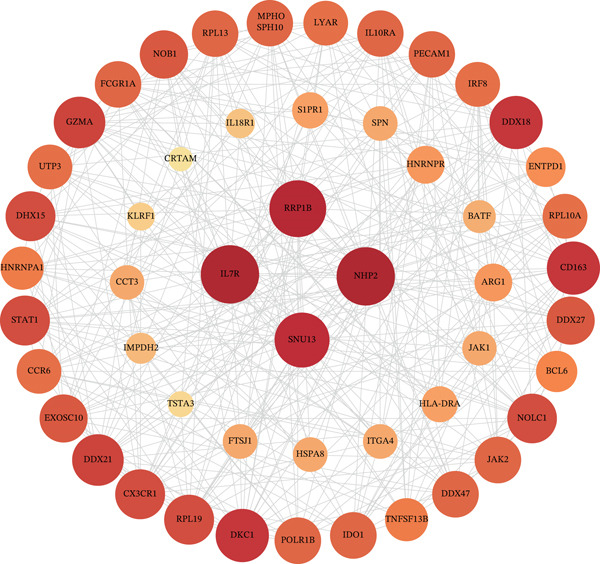
(c)

**Figure:**
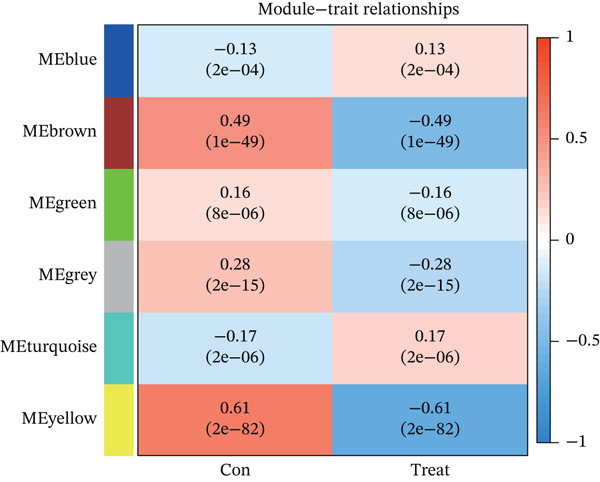
(d)

**Figure:**
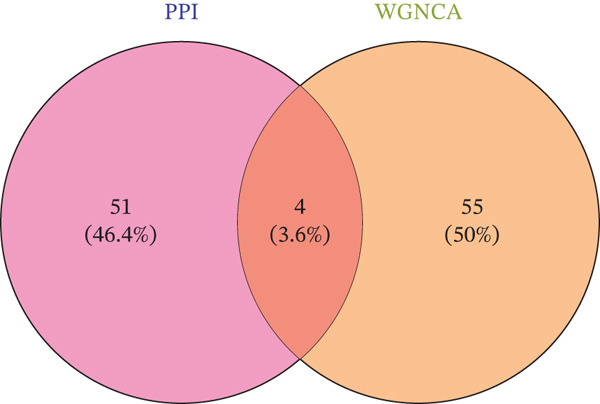
(e)

### 3.3. Machine Learning Analysis of Hub Genes

Boruta and SVM‐RFE algorithms identified DDX24, KCNA3, and NCL as having a strong diagnostic performance for distinguishing patients from controls (Figure 3a,b). DDX24 demonstrated the highest feature importance ranking. Univariate Cox regression revealed potential prognostic significance for GZMM, KCNA3, and DDX24 (Figure 3c). Multivariate analysis (stepwise Cox and LASSO regression) identified GZMM as an independent prognostic factor (Figure 3d,e). Thus, DDX24 is a key determinant for sepsis susceptibility, whereas GZMM is a critical prognostic marker.

Figure 3Machine learning identification and validation of core prognostic genes. (a) Boruta feature importance ranking. Green boxplots represent genes confirmed as important by the Boruta algorithm. Blue boxplots represent tentative hits or algorithm‐defined thresholds. Features positioned higher and further right exhibit greater importance. (b) SVM‐RFE error rate curve. The minimum point on the curve indicates the optimal feature subset size yielding the lowest error rate. The right panel ranks gene importance within the final SVM model. (c) Forest plots of univariate (upper panel) and stepwise multivariate (lower panel) Cox proportional hazards regression analyses for core genes. Genes positioned left of the vertical line (HR < 1) are protective factors; genes positioned right of the line (HR > 1) are risk factors for sepsis prognosis. (d) Lasso regression cross‐validation curve. The minimum point on the mean squared error (MSE) curve indicates the optimal lambda value. The top number indicates the corresponding number of nonzero coefficients at that lambda. (e) Lasso coefficient profiles. The vertical dashed line marks the optimal lambda value selected by cross‐validation. Genes with nonzero coefficients at this lambda are considered influential for sepsis prognosis.

**Figure:**
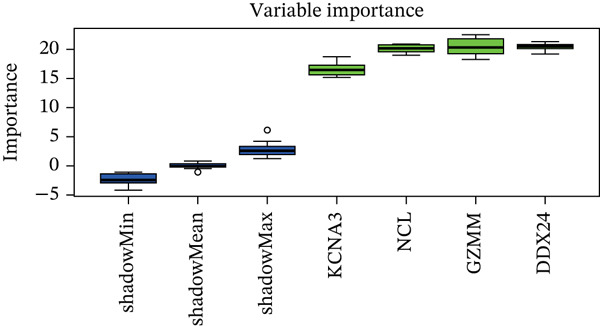
(a)

**Figure:**
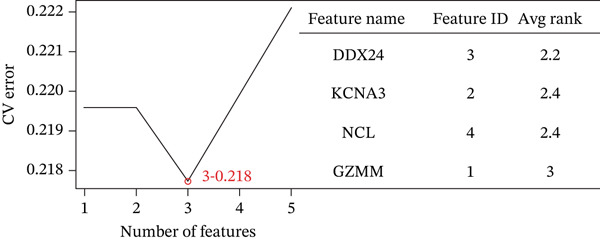
(b)

**Figure:**
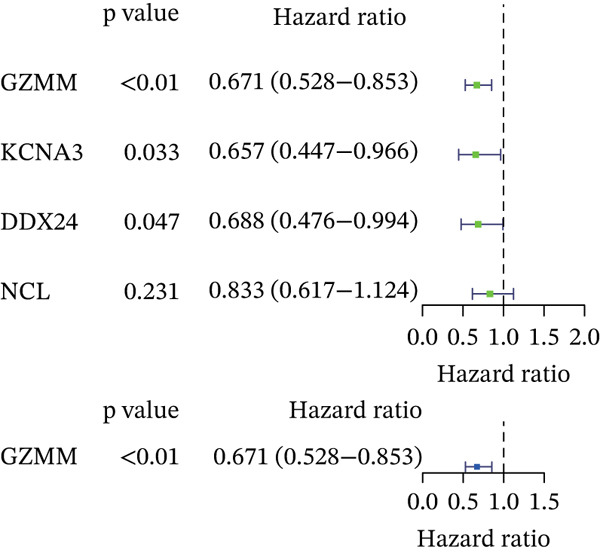
(c)

**Figure:**
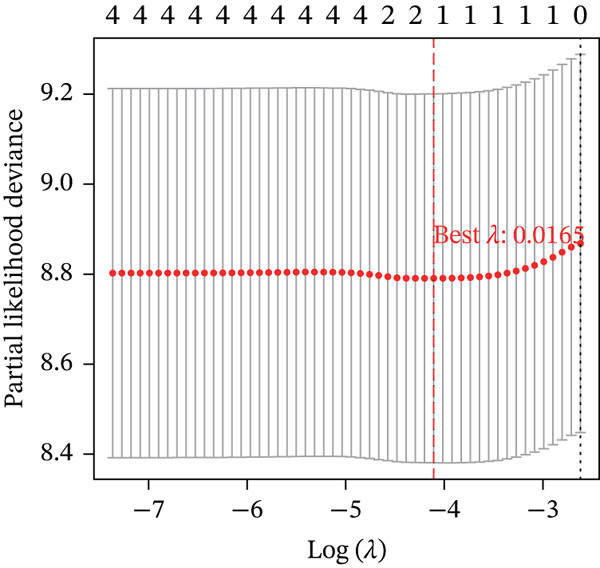
(d)

**Figure:**
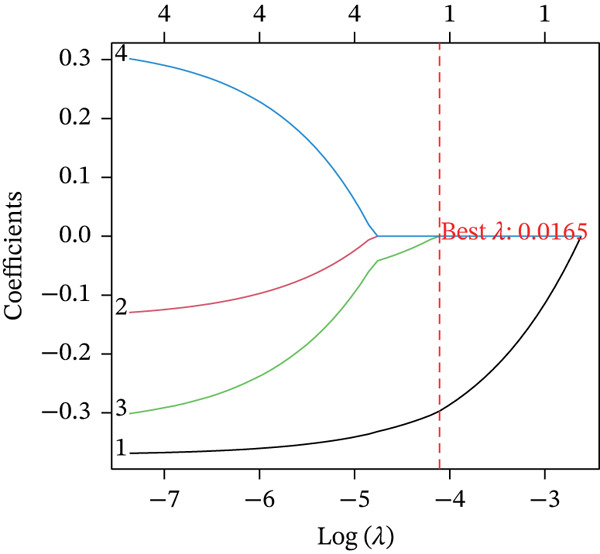
(e)

### 3.4. Validation of Hub Genes

ROC curve analysis using validation cohorts (GSE28750 and GSE95233) demonstrated excellent diagnostic performance for all four hub genes (AUC > 0.8; Figure 4a,b). qRT‐PCR validation data were analyzed using the Wilcoxon rank‐sum test due to nonnormal distribution. Stratification based on median expression revealed that only GZMM expression was significantly associated with patient outcomes (KM curves, log‐rank *p* < 0.05; Figure 4c). qRT‐PCR provided preliminary clinical verification of the significant downregulation of all four hub genes in sepsis patients from the local cohort (Figure 4d). Correlation analysis showed a significant negative association between GZMM expression and APACHE II scores (*p* < 0.05; Figure 4e,f, Table S3). Spearman correlation analysis was corrected for multiple testing using the BH method (*q* < 0.05), validating GSE65682 survival results. These findings comprehensively verify the clinical relevance of these genes, highlighting the adverse prognostic implications of decreased GZMM expression.

Figure 4Validation of core gene expression and clinical relevance. (a–b) Receiver operating characteristic (ROC) curves for core genes in the GSE95233 and GSE28750 datasets, respectively, distinguish sepsis patients from healthy controls. Higher area under the curve (AUC) values indicate greater diagnostic power. (c) Kaplan–Meier (KM) survival curves for core genes in the GSE65682 dataset. A higher curve position indicates a better survival probability for the corresponding group. (d) Validation of core gene expression by quantitative reverse transcription PCR (qRT‐PCR). (e) Scatter plots depicting the correlation between core gene relative expression levels and APACHE II scores.

**Figure:**
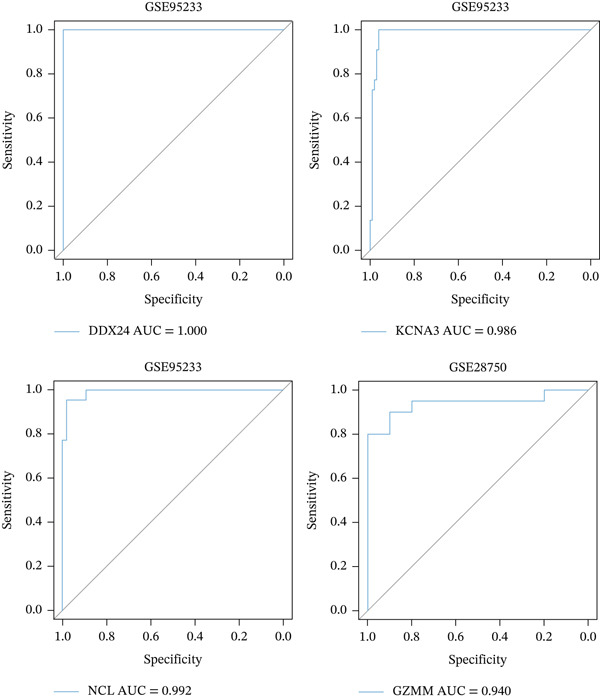
(a)

**Figure:**
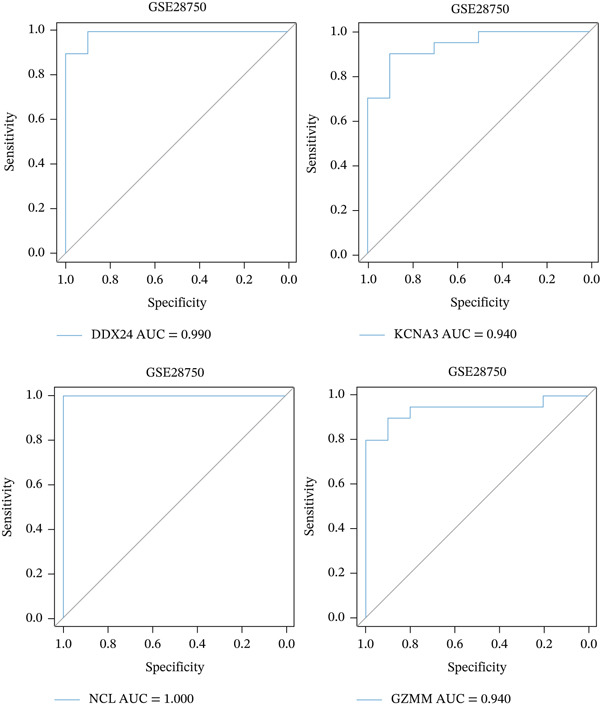
(b)

**Figure:**
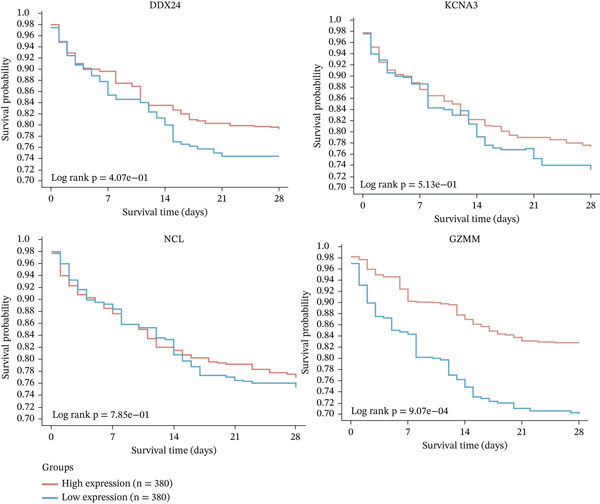
(c)

**Figure:**
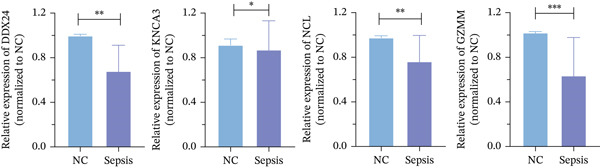
(d)

**Figure:**
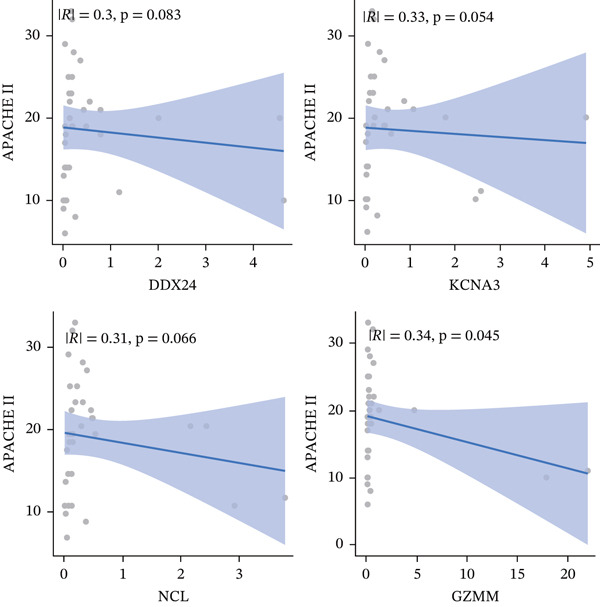
(e)

### 3.5. Functional Analysis of Core Genes

Single‐gene GSEA revealed distinct functional patterns. DDX24 is associated with ribosomal functions, immune responses, and signal transduction (Figure 5a,b). GZMM demonstrated predominant involvement in immune response regulation and ribosomal processes (Figure 5c,d). Immune infiltration analysis showed significant alterations in sepsis: decreased resting NK cells, resting mast cells, and CD8+ T cells; increased memory B cells, gamma delta T cells, and M0 macrophages (Figure 5e,f). Immune cell infiltration differences between groups were analyzed using the Wilcoxon rank‐sum test, with BH correction for multiple testing (*q* < 0.05). Correlation analysis revealed significant positive correlations between hub genes and activated dendritic cells, activated NK cells, and CD8+ T cells (*p* < 0.01), and significant negative correlations with eosinophils, M1 macrophages, and plasma cells (*p* < 0.05). GZMM exhibited the strongest positive correlation with CD8+ T cell infiltration (*r* = 0.68, *p* = 2.3 × 10^−15^; Figure 5g). All Spearman correlation analyses between hub genes and immune cell infiltration were corrected for multiple testing using the BH method (*q* < 0.05). This result was further supported by the transcript level correlation analysis between hub genes and canonical immune cell‐specific marker genes in GEO datasets (GSE65682 and GSE95233), which confirmed a significant positive correlation between GZMM expression and CD8+ T cell signature genes (CD8A and CD8B). Meanwhile, the scRNA‐seq data of this study independently verified that GZMM and DDX24 are predominantly expressed in CD8+ T cells and NK cells, and their expression levels are closely associated with the abundance of these cell populations, consistent with the CIBERSORT infiltration analysis results. Transcriptional regulatory network analysis identified 10 core TFs (e.g., ATF1, CREB3L1, and CREM) regulating ≥ 3 hub genes and 60 conserved miRNAs (e.g., hsa‐let‐7a‐5p, hsa‐let‐7b‐5p, and hsa‐let‐7c‐5p) targeting all four hub genes (Figure 5h, Figure S3e). These core TFs are key regulators of immune cell activation and inflammatory cytokine secretion, and the conserved let‐7 family miRNAs are widely involved in the regulation of innate and adaptive immune responses in sepsis, their targeted regulation of hub genes may be an important molecular mechanism mediating immune dysfunction in sepsis. Drug prediction yielded 25 FDA‐approved candidates: 4 targeting hub genes directly, and 21 targeting core TFs (Figure 5i), highlighting translational value.

Figure 5Functional analysis of core genes. (a–d) gene set enrichment analysis (GSEA) enrichment plots for DDX24 (GO: A, KEGG: B) and GZMM (GO: C, KEGG: D). A positive normalized enrichment score (NES, peak position > 0) indicates a positive correlation with the pathway; a negative NES (peak position < 0) indicates a negative correlation. The middle section ranks genes within the pathway based on their correlation with the core gene (strongest correlation on the left). The bottom curve depicts the running enrichment score. (e) Stacked bar plot showing the relative proportions of immune cell types inferred by deconvolution analysis. Bar length represents the estimated proportion of each cell type. (f) Boxplots comparing immune cell infiltration scores between sepsis patients and healthy controls (Wilcoxon test). (g) Spearman correlation heatmap between immune cell infiltration scores and the expression levels of key core genes (DDX24, GZMM). Red indicates a positive correlation, blue indicates a negative correlation; darker shades represent stronger correlations. (h) Simplified transcriptional regulatory network of core genes. Blue circles represent core genes, green triangles represent targeting miRNAs, and orange diamonds represent transcription factors (TFs).  ^∗^ *p* < 0.05,  ^∗∗^ *p* < 0.01,  ^∗∗∗^ *p* < 0.001. (i) Drug prediction analysis showing 25 FDA‐approved candidate drugs, including 4 targeting hub genes directly and 21 targeting core transcription factors (TFs).

**Figure:**
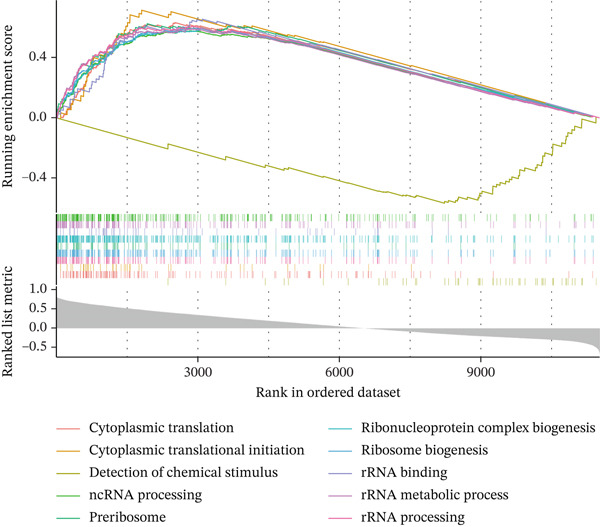
(a)

**Figure:**
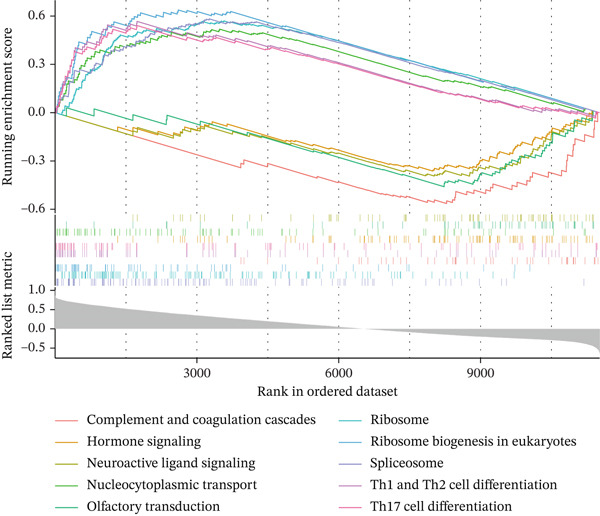
(b)

**Figure:**
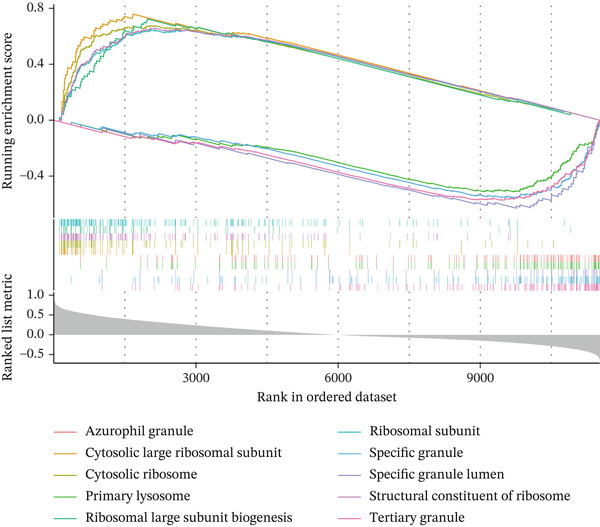
(c)

**Figure:**
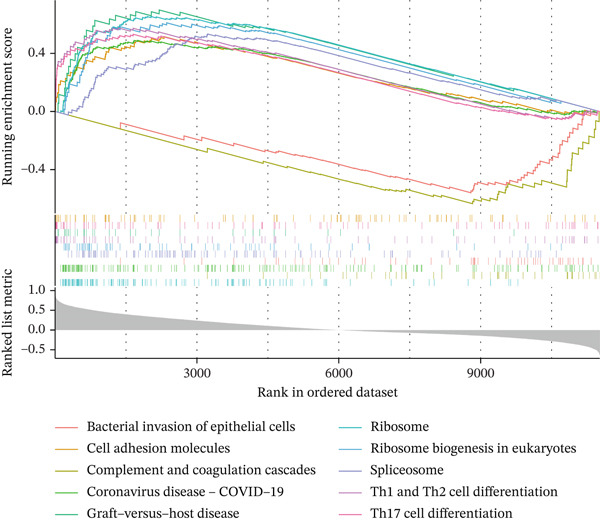
(d)

**Figure:**
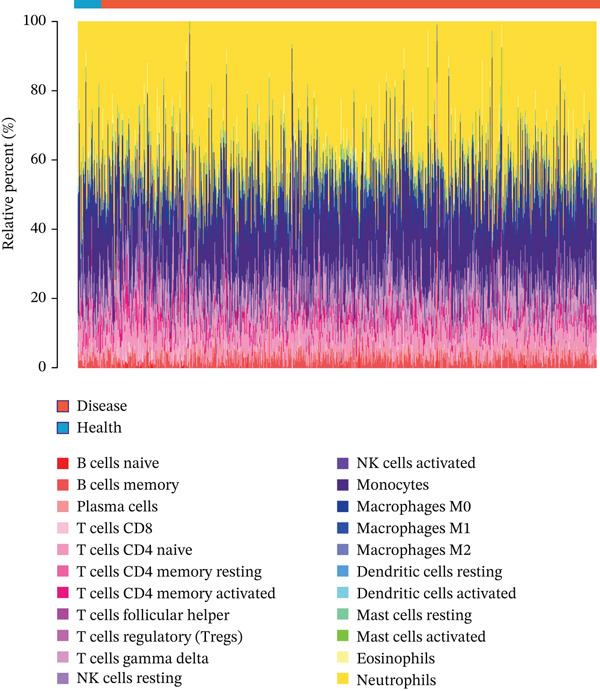
(e)

**Figure:**
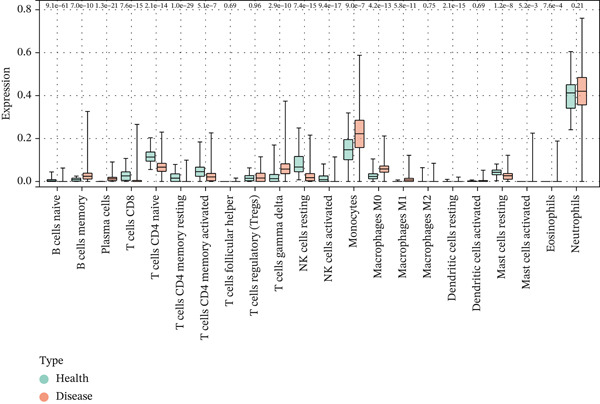
(f)

**Figure:**
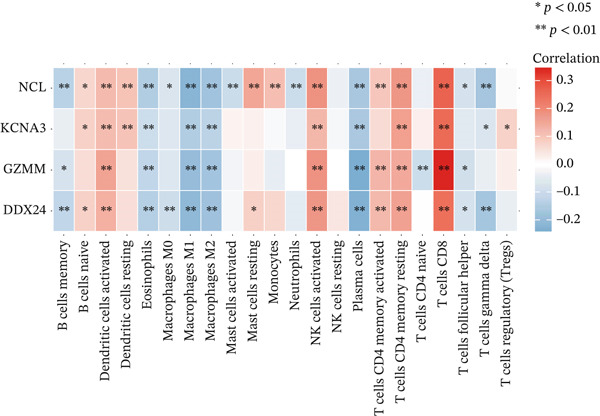
(g)

**Figure:**
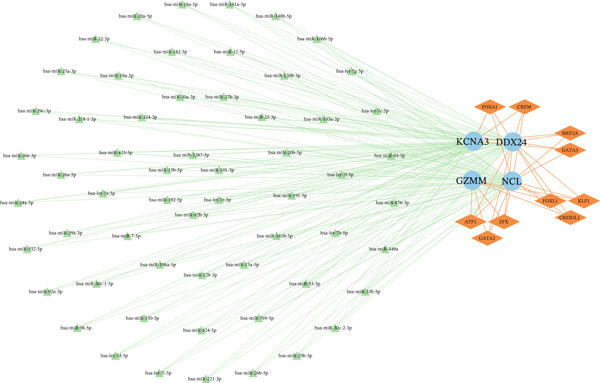
(h)

**Figure:**
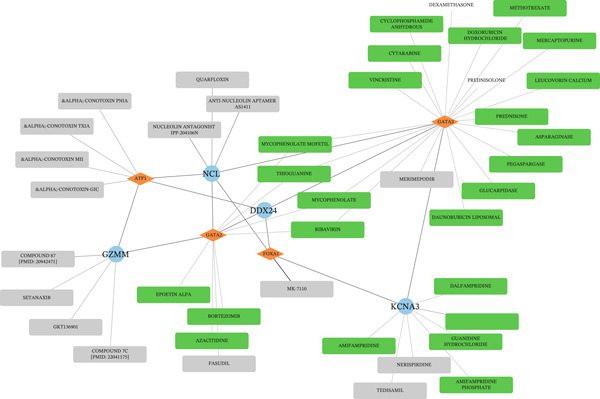
(i)

### 3.6. Single‐Cell Analysis of Core Genes

scRNA‐seq analysis (Figure S4) showed GZMM and DDX24 predominantly expressed in lymphocyte populations (T cells, NK cells, and B cells), especially T cells (Figures 6a, 6b, and 6c). Monocyte proportion was significantly suppressed in patients. Granulocyte and platelet proportions were abnormally high in deceased patients versus survivors. T cell and NKT cell proportions showed a continuous downward trend across Healthy, T6_Survive, T6_Death, T0_Survive, and T0_Death groups (Figure 6d), indicating higher mortality risk associated with lower T/NKT cell proportions. DDX24 expression was significantly lower in patients than controls (Figure 6e). Within the disease group (Figure 6i), T0 deceased patients had significantly lower DDX24 expression in positive cells than T6 deceased patients. At T0, deceased patients showed significantly lower expression than survivors. Conversely, at T6, deceased patients showed significantly higher DDX24 expression than survivors. Unlike DDX24 (Figure 6f,j), GZMM showed no significant differences between T0 deceased versus T6 deceased or T0 deceased versus T0 survivors. However, GZMM expression in positive cells was significantly lower in T6 deceased patients than in T6 survivors (Figure 6j). These results demonstrate as follows:

1. T cell and NKT cell proportions are significantly reduced in sepsis patients and decrease further with increasing mortality risk.
2. DDX24 and GZMM are mainly expressed in T cells and NK cells.
3. DDX24 expression is significantly reduced primarily during early infection stages.
4. GZMM expression level primarily affects patient survival status in later infection stages. All scRNA‐seq gene expression and cell proportion comparisons were analyzed using the Wilcoxon rank‐sum test, with BH correction for multiple testing (*q* < 0.05).

Figure 6Single‐cell RNA sequencing (scRNA‐seq) analysis. (a) *t*‐Distributed stochastic neighbor embedding (t‐SNE) plot showing cell clusters (left) and annotated cell types (right). (b, c) *t*‐SNE plots visualizing DDX24 (b) and GZMM (c) expression across cell clusters. Gray dots represent negative cells; red dots represent positive cells. (d) Violin plots showing the proportion of each cell type across patient groups. (e, f) Violin plots comparing DDX24 (e) and GZMM (f) expression levels between sepsis patients and healthy controls across all cells (Wilcoxon test). (g, h) Violin plots comparing DDX24 (g) and GZMM (h) expression levels between sepsis patients and healthy controls within specific cell clusters (Wilcoxon test). (I, J) Boxplots comparing the proportion of DDX24‐positive (i) and GZMM‐positive (j) cells between patients with different survival statuses (Wilcoxon test).  ^∗^ *p* < 0.05,  ^∗∗^ *p* < 0.01,  ^∗∗∗^ *p* < 0.001.

**Figure:**
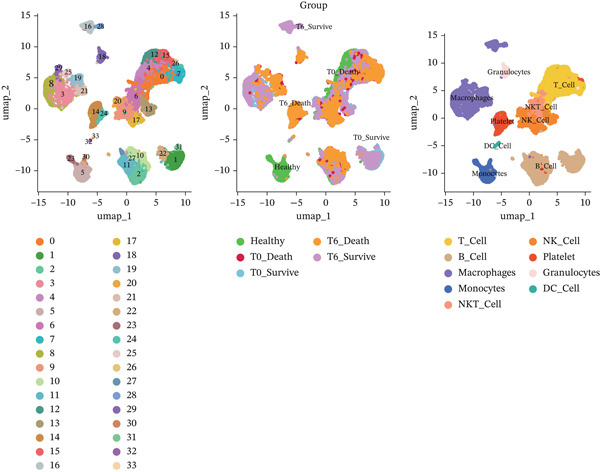
(a)

**Figure:**
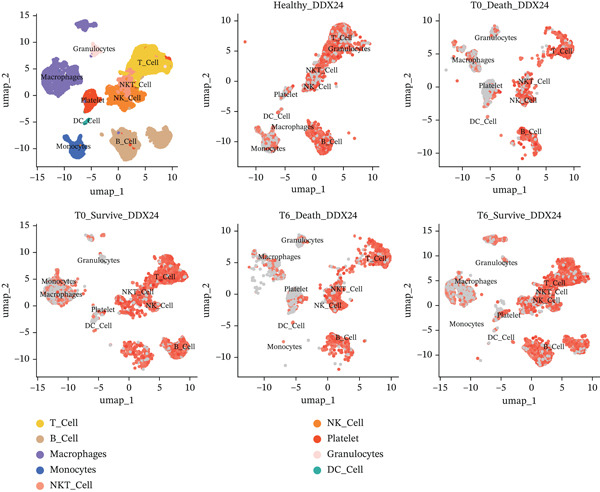
(b)

**Figure:**
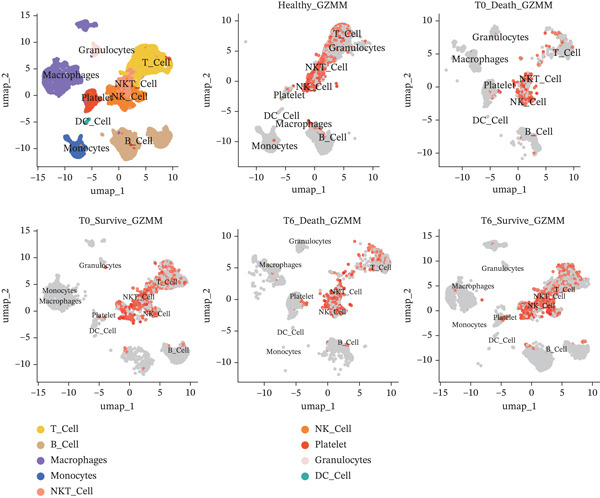
(c)

**Figure:**
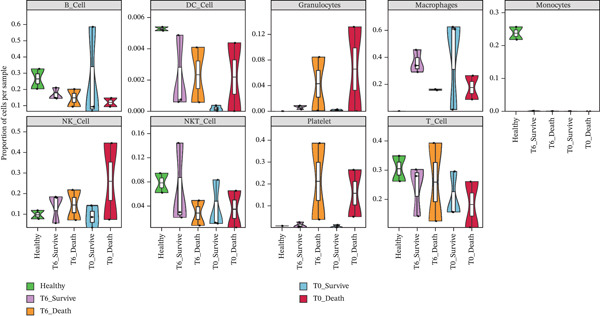
(d)

**Figure:**
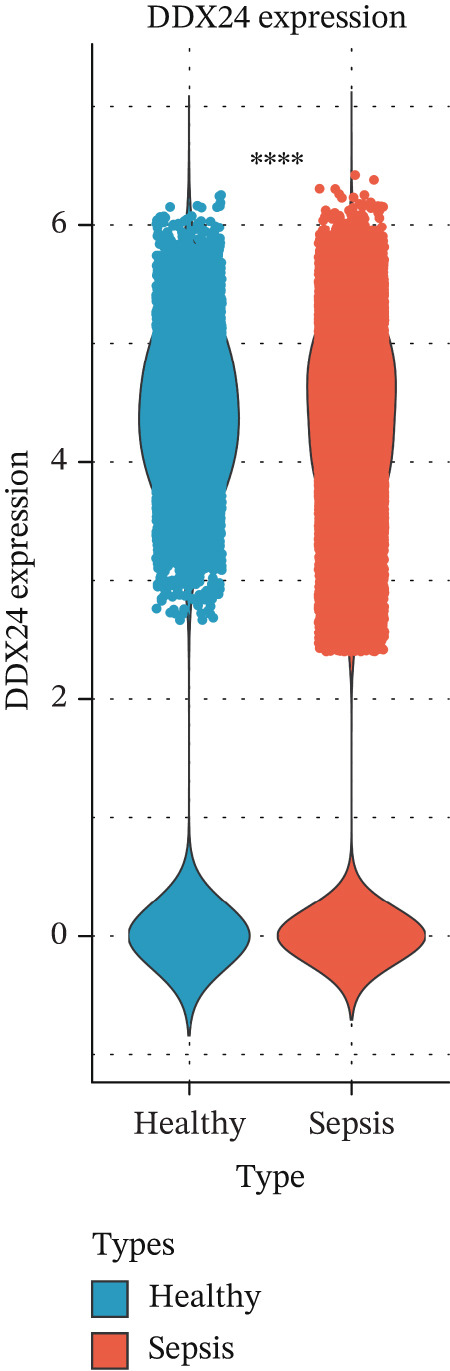
(e)

**Figure:**
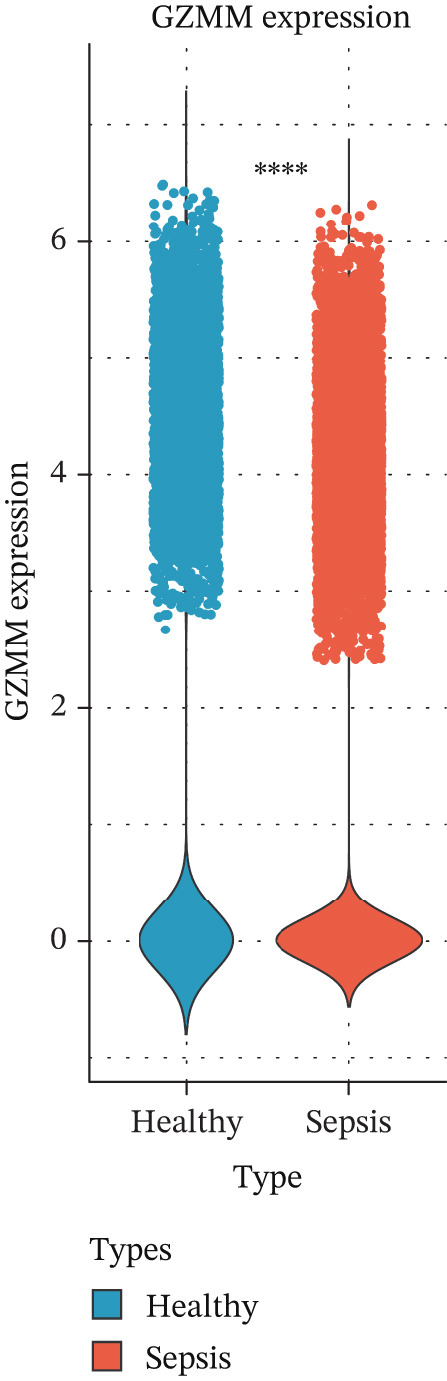
(f)

**Figure:**
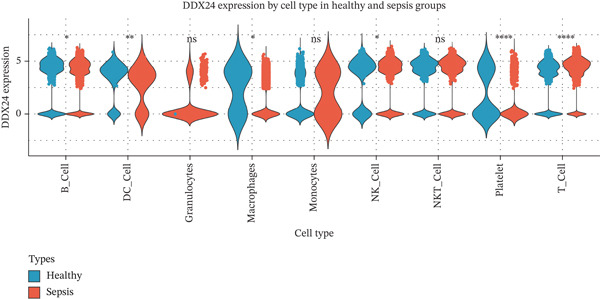
(g)

**Figure:**
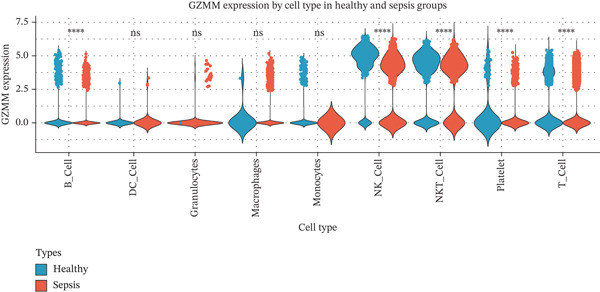
(h)

**Figure:**
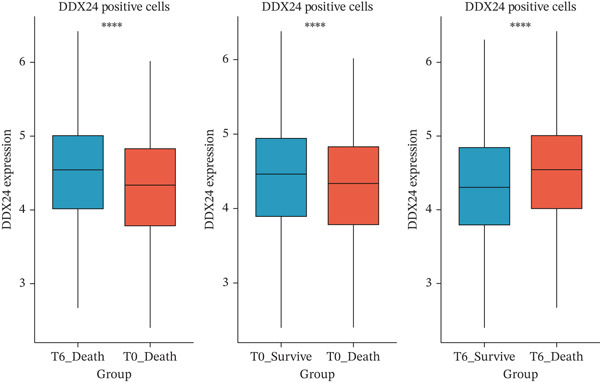
(i)

**Figure:**
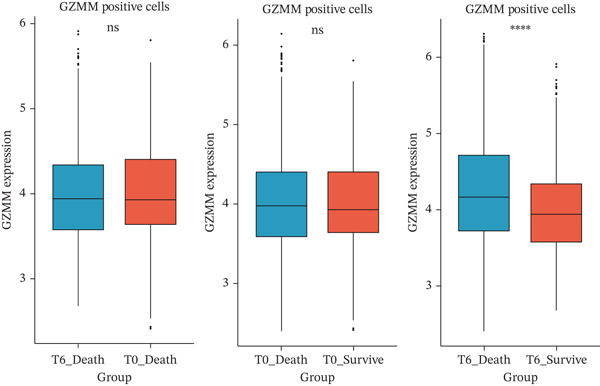
(j)

## 4. Discussion

Severe immunopathological complexity of sepsis as well as its high rate of mortality [22] is still a serious clinical problem. In spite of progress in biomarker research, early and precise diagnostic and prognostic assessment are hard to achieve. Applying a combination of bioinformatics and clinical verification, we explored significant regulator genes in sepsis, with special emphasis on temporally regulated GZMM and DDX24 expression and function in T‐cell–mediated immunity.

Expression of granzyme M (GZMM) in NK cells, NKT cells, and subsets of CD8+ T cells also has great prognostic significance [23, 24]. We found a negative correlation between GZMM expression levels and APACHE II scores (*r* = –0.52, *p* < 0.01), and downregulation of GZMM correlated with the decreased levels of T cells and NK cells identified by single‐cell analysis. Our functional enrichment analysis revealed that GZMM is a key regulator of the TCR‐signaling pathway, and its expression was strongly positively correlated with CD8+ T cell infiltration. The downregulation of GZMM may directly inhibit TCR‐mediated activation and proliferation signals of CD8+ T cells, leading to the functional exhaustion of effector CD8+ T cells and impaired cytotoxicity against pathogens. Meanwhile, the reduced GZMM expression formed a positive feedback loop with the decreased proportion of resting NK cells and CD8+ T cells in sepsis patients, further weakening the adaptive immune response and exacerbating the formation of an immunosuppressive microenvironment in late sepsis. Consistent with the role played by GZMM in enhancing the downstream effects of TLR4 activation such that it facilitates the production of proinflammatory cytokines (e.g., IL‐1*β* and TNF‐*α*) and potentiates inflammation [25, 26], downregulation of GZMM during late sepsis may indicate exhaustion of T/NK cells, which may compromise immunity. Indeed, overexpression of GZMM in platelets was shown in sepsis patients by Chennareddy et al.; increased GZMM may lead to immune suppression via facilitating apoptosis in CD4+ T cells [27, 28]. In our single‐cell data sets, expression levels of GZMM are lower in nonsurvivors versus survivors at T6 (infection stage: late), demonstrating that varying levels of GZMM expression may be useful markers in determining the functional status of immune cells and predicting outcomes.

DDX24 (DEAD‐box helicase 24) was significantly downregulated in sepsis patients [29, 30], especially at the beginning of infection in nonsurvivals. Functionally, DDX24 controls RNA turnover/ribosomal biosynthesis and participates in stress response/immune modulation [31, 32]. It repressed RIG‐I‐like receptor (RLR) signaling pathways and thus the innate immune response [33, 34]. It also played a regulatory role in cell growth/senescence by blocking EP300‐mediated TP53 acetylation [35]. High DDX24 expression in gastric cancer was associated with lower numbers of infiltrating CD8+ T cells as well as less expression of immune checkpoint molecules [36], which supports its possible role in sepsis‐induced immunosuppression. Despite DDX24 being a good candidate for diagnosing biomarker (AUC > 0.8), there was no association between the level of DDX24 expression and prognosis. One possible explanation for this discrepancy could lie in the dynamic change of DDX24 expression. For instance, its initial decrease could indicate activation of innate immunity and uncontrolled proliferation of T cells. Subsequent elevation of DDX24 may lead to exhaustion of T cells. Further research will need to determine DDX24′s specific molecular targets within the sepsis immune microenvironment.

Despite their lack of significant prognostic significance, both KCNA3 and NCL have diagnostic implications as well as some relevance to immune pathways. KCNA3 is a voltage‐gated potassium channel induced in activated T cells and may regulate T cell activity through alteration of membrane potential [37, 38]. NCL participates in viral infection processes, negatively correlates with sepsis‐related genes [39]; and might help regulate immunity through virus–host interaction.

Drug prediction revealed 25 putative drug candidates consisting of GATA2‐targeting drugs (e.g., azacitidine and bortezomib), which have potential to mitigate immune dysregulation through modification of GZMM and DDX24 expression. The immunosuppressant MYCOPHENOLATE, inhibiting T/B cell proliferation, might attenuate excessive inflammation in early sepsis [40]. Azacitidine modulates the activity of TFs such as GATA2 by inhibiting DNA methyltransferases, whereas bortezomib suppresses excessive inflammatory responses via blocking NF‐*κ*B activation and regulating T/NK cell function. We hypothesize that stage‐specific modulation of DDX24/GZMM and their regulatory TFs could restore the innate‐adaptive immune balance in sepsis: Upregulating DDX24 may alleviate the early inflammatory storm by inhibiting overactivated RLR signaling, and targeting GZMM can reverse late T/NK cell exhaustion; candidate drugs targeting core TFs can fine‐tune the expression of these hub genes, thus breaking the inflammation‐immunosuppression cascade and improving sepsis outcomes.

In conclusion, the dynamic expression patterns of GZMM and DDX24 provide novel perspectives for sepsis diagnosis and prognosis assessment. Their roles in regulating T cell function and immune homeostasis offer promising research avenues, deepening our understanding of sepsis pathogenesis and establishing a theoretical foundation for precision medicine approaches. This study has several notable limitations. First, integrating bulk and single‐cell RNA‐seq data has inherent pitfalls: Bulk sequencing reflects average gene expression in heterogeneous cell populations, masking cell‐type‐specific dynamics, whereas differences in sample processing and analytical pipelines between the two technologies may lead to inconsistent inferences. Second, our bioinformatics analyses are purely correlative, and no causal inferences can be drawn between hub gene expression and sepsis immune dysfunction, as unmeasured confounding factors may drive both gene expression changes and disease progression. Third, the qRT‐PCR validation cohort is severely underpowered (35 sepsis patients, 6 healthy controls) without detailed clinical demographic data, which impairs the statistical validity, stratification analysis and generalizability of our clinical findings, and increases the risk of Type II error. Fourth, the specific molecular mechanisms by which GZMM and DDX24 regulate T/NK cell function and sepsis immune dysregulation remain uncharacterized. To address these limitations, future functional experiments are essential, including in vitro knockdown/overexpression of GZMM and DDX24 in primary T/NK cells to assess cell activation, cytotoxicity and cytokine secretion under septic stimuli, and in vivo sepsis models with cell‐type‐specific knockout/overexpression to validate their causal roles. Additionally, larger, well‐characterized clinical cohorts are needed to replicate the diagnostic and prognostic value of GZMM and DDX24, and integrated single‐cell multiomics coupled with functional validation will further characterize their regulatory networks in sepsis, facilitating targeted therapeutic strategies.

## Author Contributions

Yi Zhang: conceptualization, formal analysis, writing—original draft; Liang Tang: formal analysis, data curation, validation; Juan Wu: formal analysis, investigation, visualization; Lin Yang: data curation, validation, writing—original draft; Wen Liu: investigation, resources, visualization; Yi Liang: resources, methodology, writing—review and editing; Jianfang Han: methodology, investigation, writing—review and editing; Shuang He: resources, data curation, writing—review and editing; Yulian Yang: conceptualization, supervision, funding acquisition, writing—review and editing, project administration. Yi Zhang, Liang Tang, Juan Wu, and Lin Yang contributed equally to this work.

## Funding

This study was supported by Deyang Science and Technology Program (2024SZY085).

## Ethics Statement

All human tissue studies were performed in accordance with the Declaration of Helsinki (2013 revision) and approved by the Ethics Committee of Southwest Medical University (Number: KY2024182). Informed consent was obtained from all study participants or their legal guardians for the collection and use of peripheral blood samples and related research data.

## Consent

Informed consent was obtained from legal guardians. The participant has consented to the submission of the case report to the journal.

## Conflicts of Interest

The authors declare no conflicts of interest.

## Supporting Information

Additional supporting information can be found online in the Supporting Information section.

## Supporting information


**Supporting Information 1** Figure S1: Supplementary results of DEG analysis between sepsis patients and healthy controls.

**Supporting Information 2** Figure S2: Supplementary results of core gene screening.

**Supporting Information 3** Figure S3: Supplementary functional analysis of core genes.

**Supporting Information 4** Figure S4: scRNA‐seq quality control (QC) and cell type annotation.

**Supporting Information 5** Table S1: Differentially expressed genes (DEGs) in GSE65682.

**Supporting Information 6** Table S2: Candidate hub genes from WGCNA.

**Supporting Information 7** Table S3: Correlation analysis of hub genes.

## Data Availability

The data that support the findings of this study are available in GEO at https://www.ncbi.nlm.nih.gov/geo/, Reference Numbers GSE65682, GSE28750, GSE95233, and GSE167363. These data were derived from the following resources available in the public domain: GSE65682, https://www.ncbi.nlm.nih.gov/geo/query/acc.cgi?acc=GSE65682; GSE28750, https://www.ncbi.nlm.nih.gov/geo/query/acc.cgi?acc=GSE28750; GSE95233, https://www.ncbi.nlm.nih.gov/geo/query/acc.cgi?acc=GSE95233; GSE167363, https://www.ncbi.nlm.nih.gov/geo/query/acc.cgi?acc=GSE167363.
