# Supplementary material for: Immunomodulatory Roles and Clinical Significance of GZMM and DDX24 in Sepsis: A Multiomics Integrative Analysis With Experimental Validation
Source: Hum Mutat. 2026 Apr 3;2026:4951633. doi: 10.1155/humu/4951633 (PMC13051877; doi:10.1155/humu/4951633)
Supplement: Supplementary file 1 — Supporting Information 1 Figure S1: Supplementary results of DEG analysis between sepsis patients and healthy controls. [file HUMU-2026-4951633-s005.docx]

**Supplementary Figure 1.** Supplementary results of DEG analysis between sepsis patients and healthy controls.


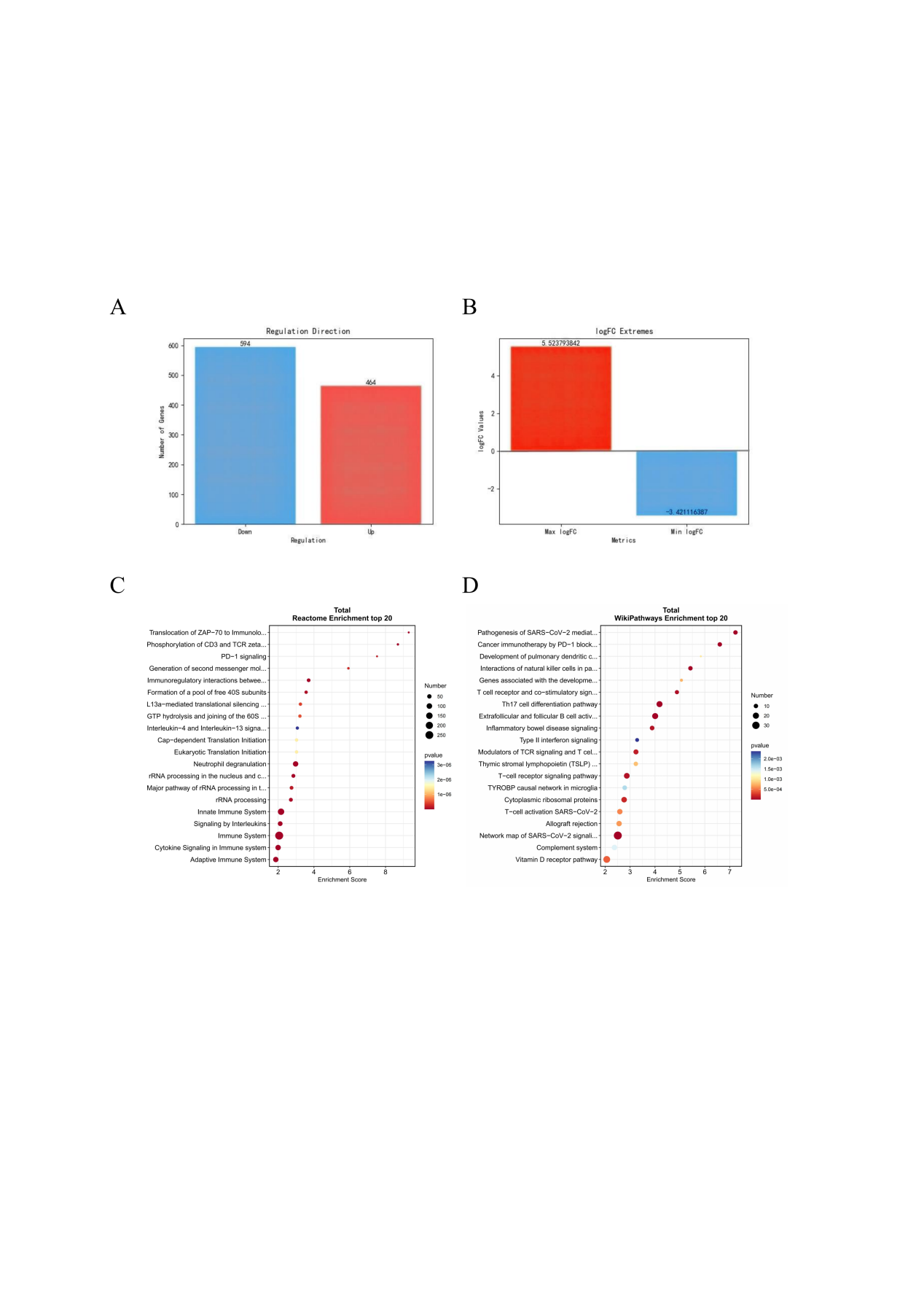


(**A**–**B**) Bar charts showing the number (**A**) and log2 fold-change (**B**) of upregulated and downregulated genes. (**C**, **D**) Bubble plots depicting Reactome (**C**) and WikiPathways (**D**) enrichment results. Bubble color intensity (red) represents the significance level -log10(*P*-value); bubble size represents the number of enriched genes.


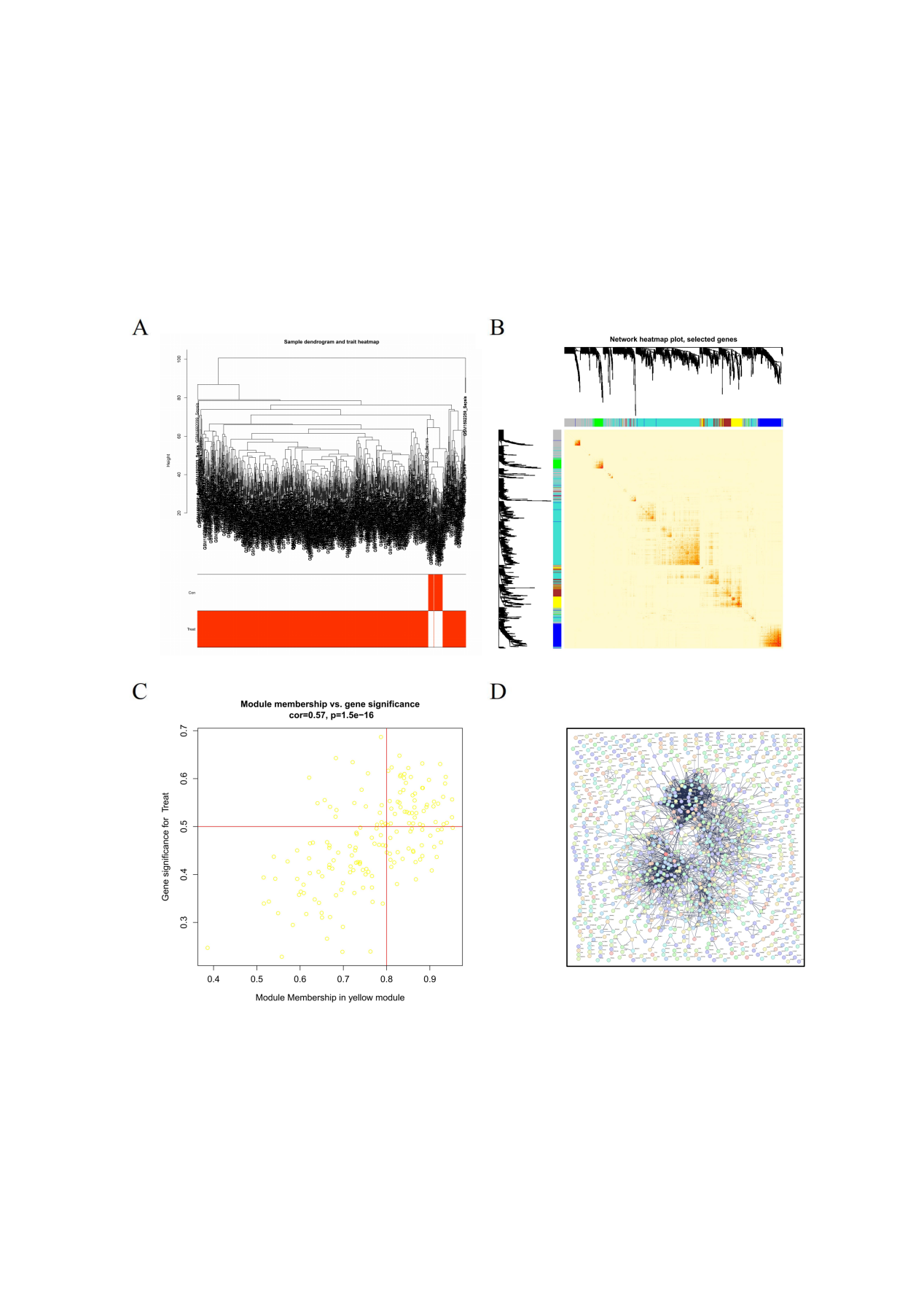


**Supplementary Figure 2.** Supplementary results of core gene screening.

(**A**) Sample dendrogram for WGCNA (top) and trait heatmap (bottom). Red indicates the presence of a specific trait in a sample. (**B**) Topological Overlap Matrix (TOM) heatmap of all analyzed genes. Lighter colors indicate lower overlap (similarity), and darker orange indicates higher overlap. (**C**) Module membership (kME) vs. gene significance (GS) scatter plots for key modules. (**D**) PPI network of differentially expressed genes.


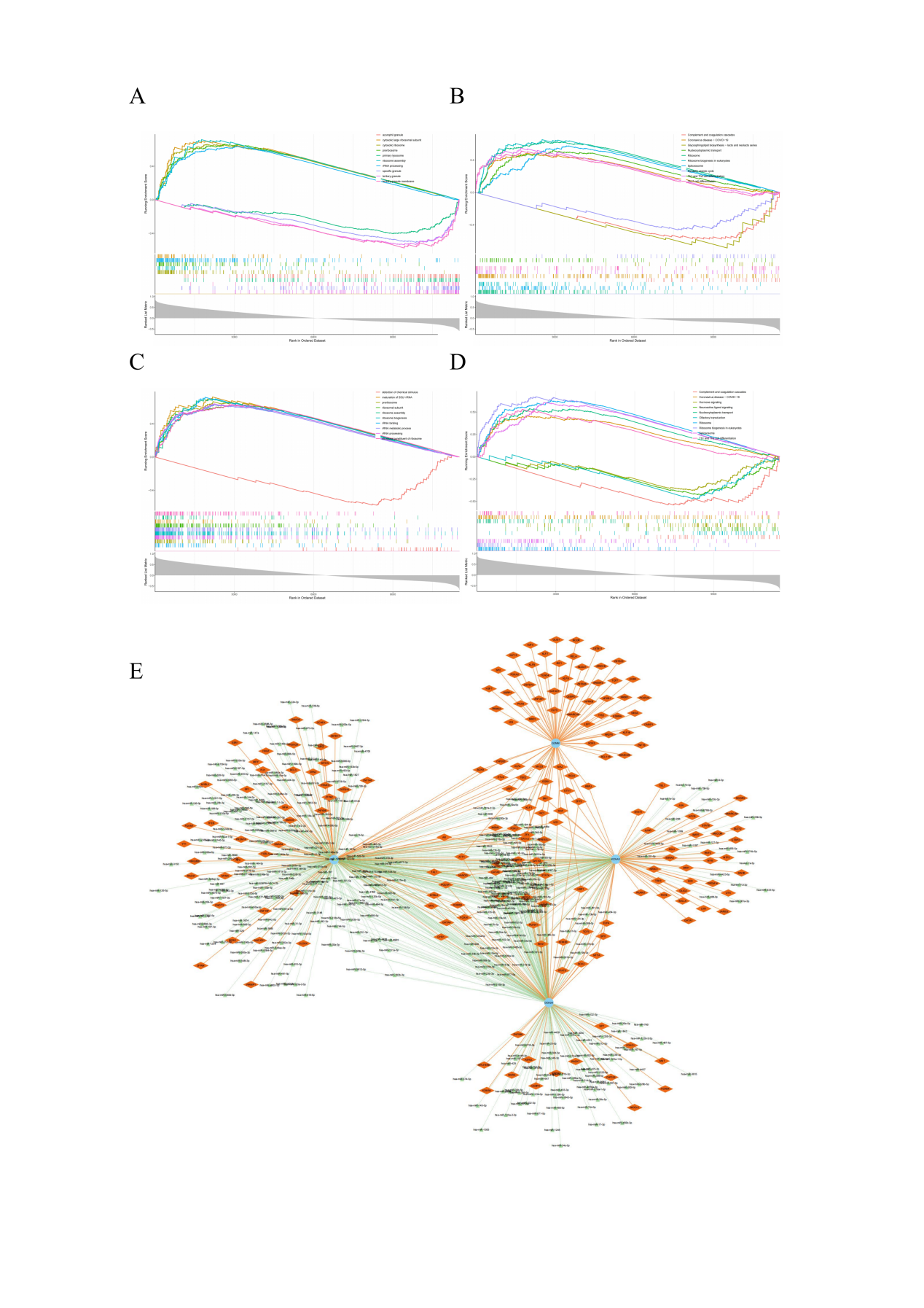


**Supplementary Figure 3.** Supplementary functional analysis of core genes.

(**A**–**D**) GSEA enrichment plots for KCNA3 (GO: A, KEGG: B) and NCL (GO: C, KEGG: D). Interpretation as in Figure 5A–D. (**E**) Comprehensive transcriptional regulatory network of core genes. Symbols as in Figure 5H.


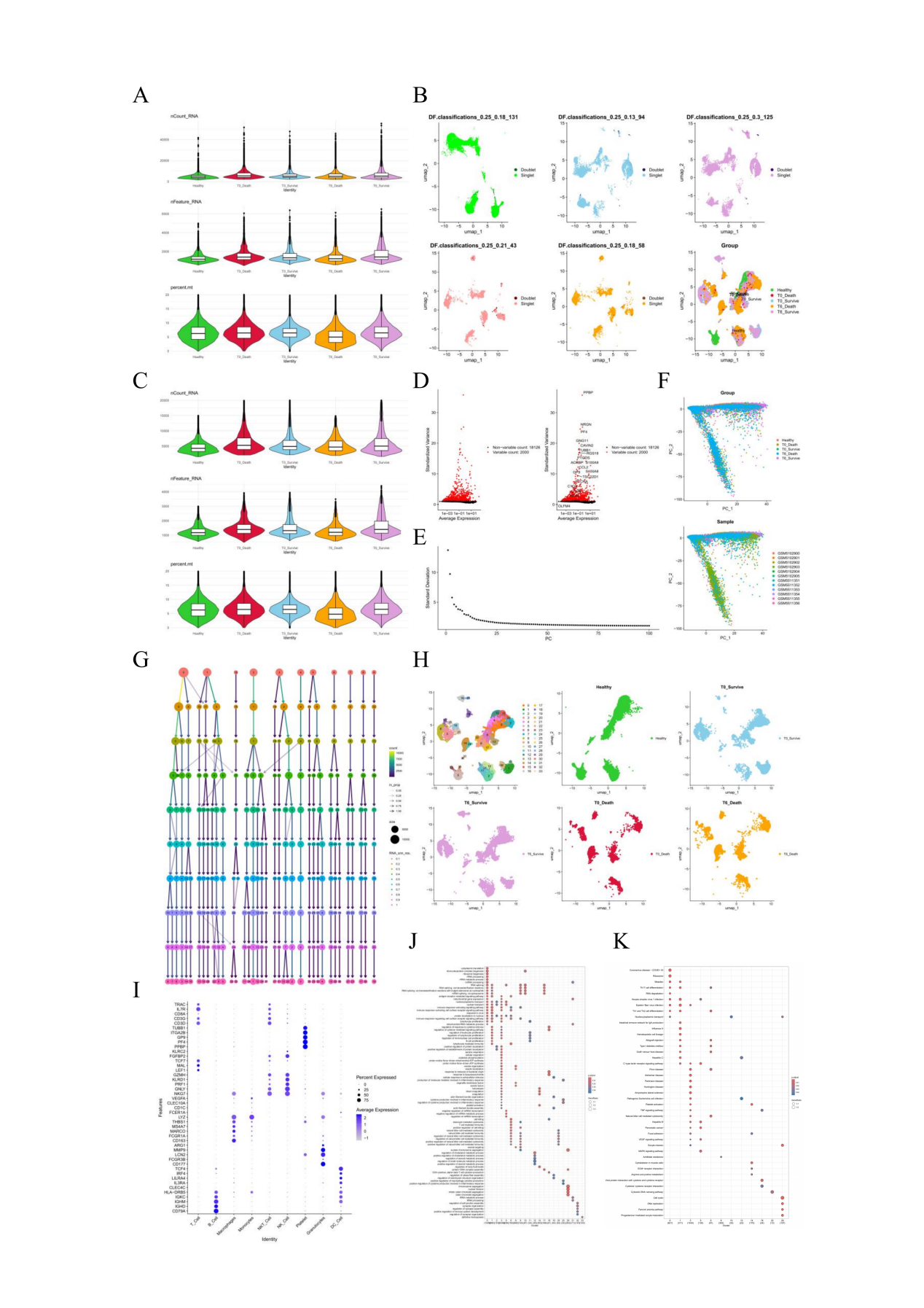


**Supplementary Figure 4.** scRNA-seq quality control (QC) and cell type annotation.

(**A**) Violin plots showing the distribution of QC metrics pre-filtering: number of genes per cell (nFeature_RNA), number of UMIs per cell (nCount_RNA), and percentage of mitochondrial genes (percent.mt). (**B**) Doublet detection UMAP plot. Darker points represent predicted doublets. (**C**) Violin plots showing QC metric distributions post-filtering. (**D**) Feature scatter plot post-normalization. (**E**) Principal component analysis (PCA) scree plot. (**F**) PCA scatter plots are colored by group (top) and sample origin (bottom). (**G**) Clustering tree at different resolution parameters. (**H**) the t-SNE plot of final cell clusters. (**I**) Bubble plot of marker genes for annotated cell types. Bubble size represents the percentage of cells expressing the marker within a cluster; color intensity (blue) represents the average expression level.
