# Supplementary figures and images for: Immunomodulatory Roles and Clinical Significance of GZMM and DDX24 in Sepsis: A Multiomics Integrative Analysis With Experimental Validation
Source: Hum Mutat. 2026 Apr 3;2026:4951633. doi: 10.1155/humu/4951633 (PMC13051877; doi:10.1155/humu/4951633)

A

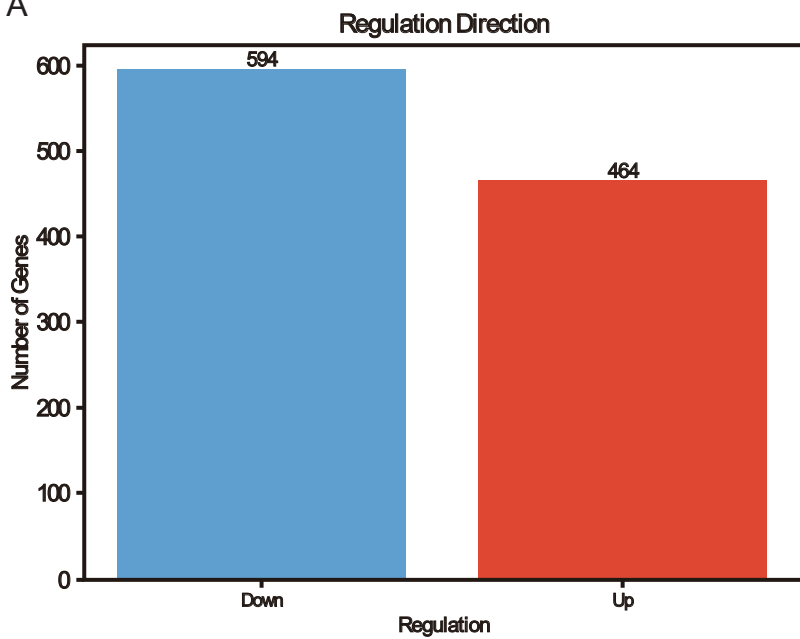

B

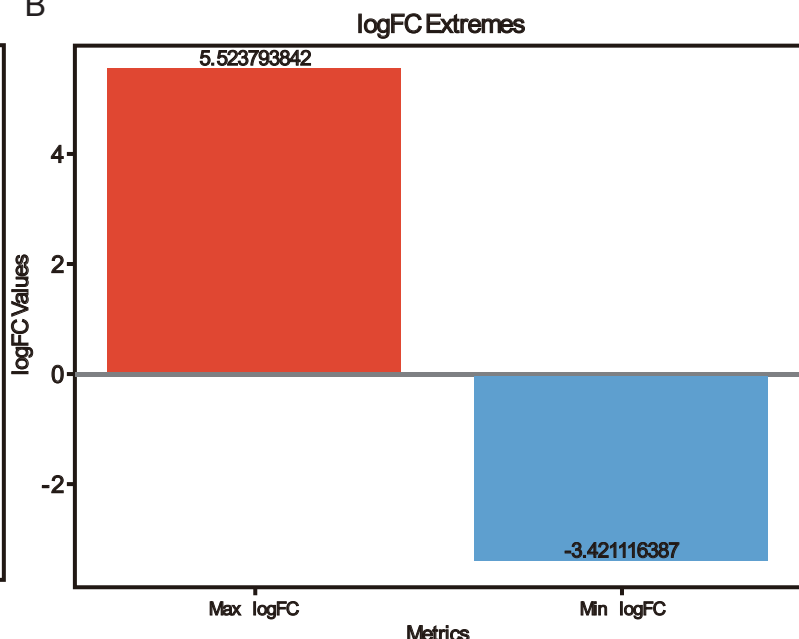

C

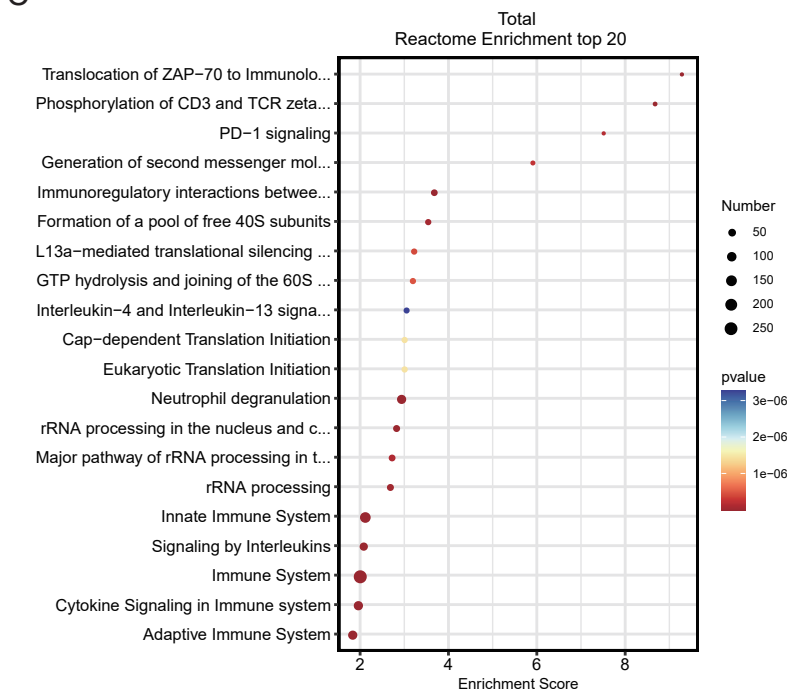

D

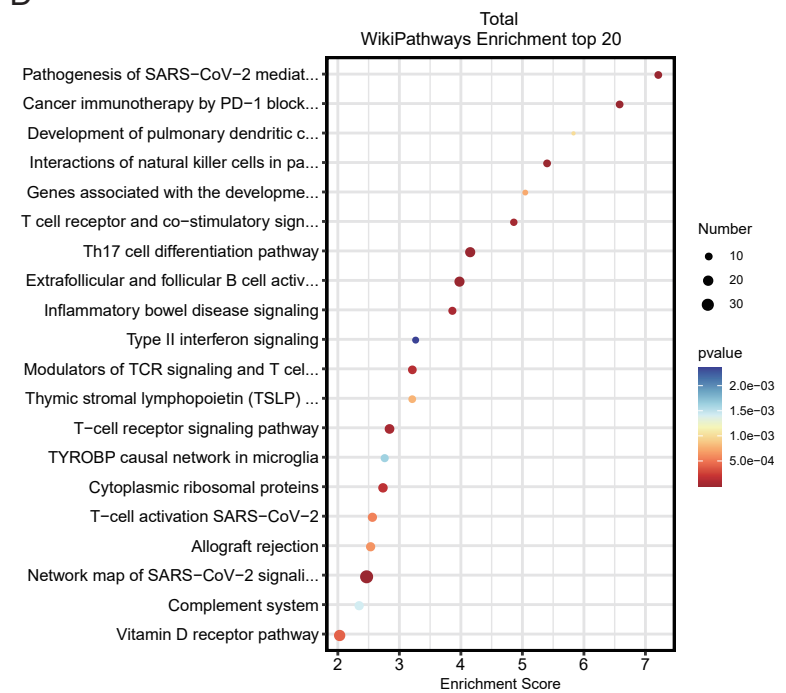

Supplement: Supplementary file 2 — Supporting Information 2 Figure S2: Supplementary results of core gene screening. [file HUMU-2026-4951633-s006.pdf]

C

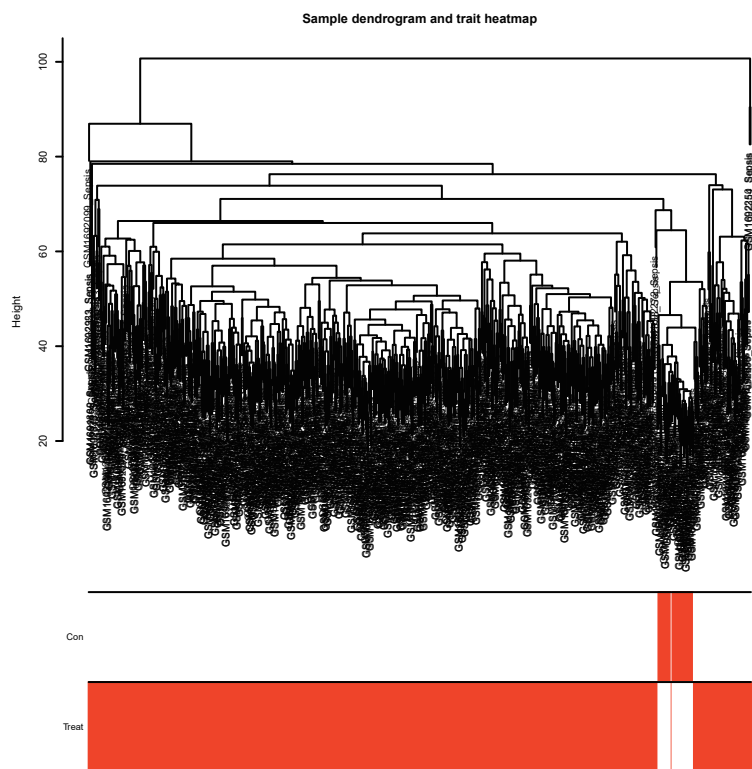

D

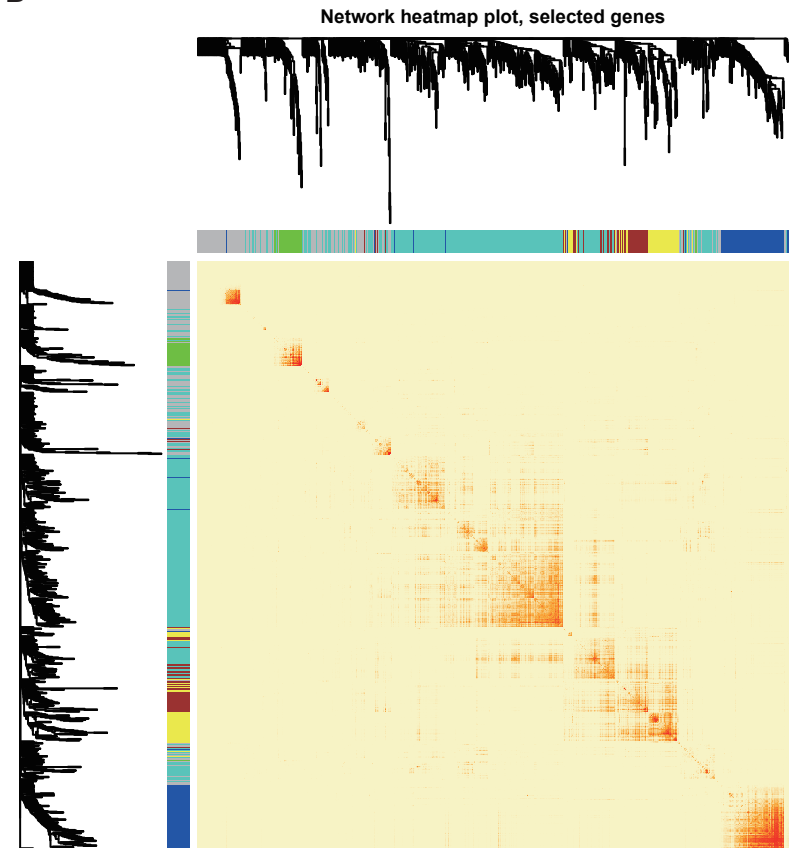

C

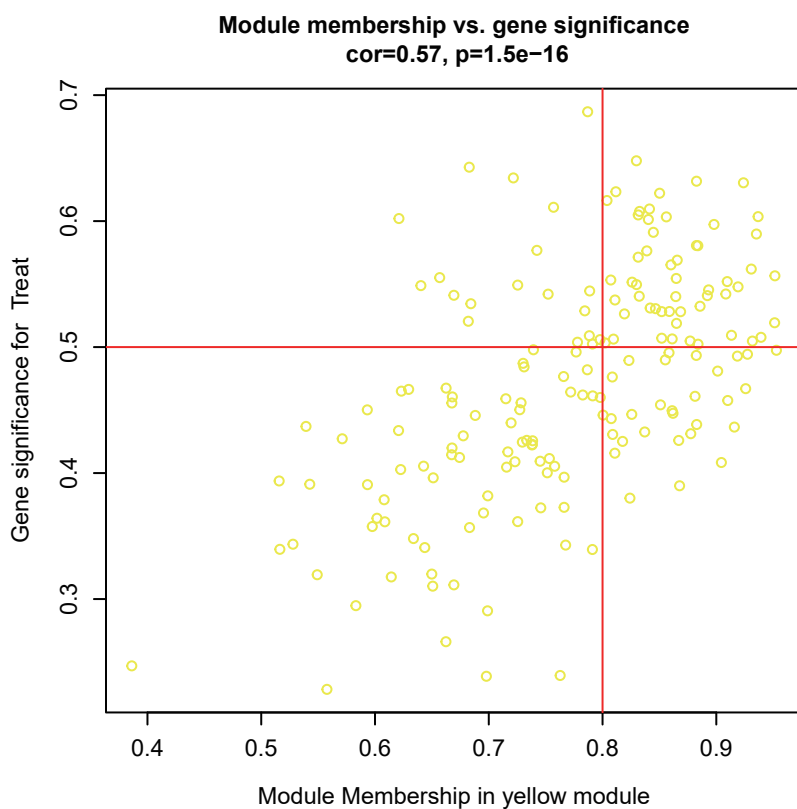

D

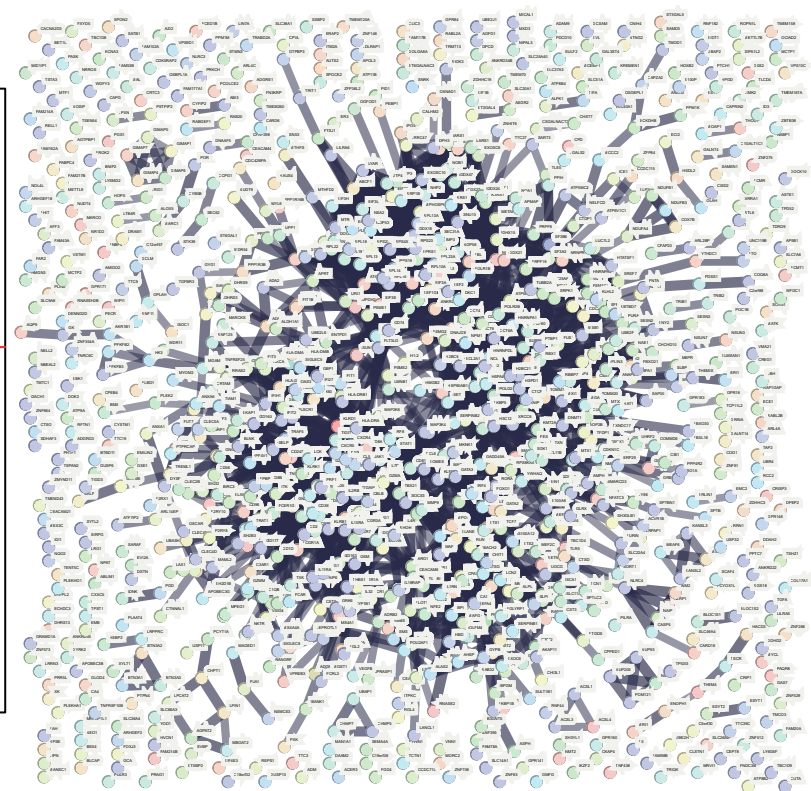

Supplement: Supplementary file 3 — Supporting Information 3 Figure S3: Supplementary functional analysis of core genes. [file HUMU-2026-4951633-s004.pdf]

E

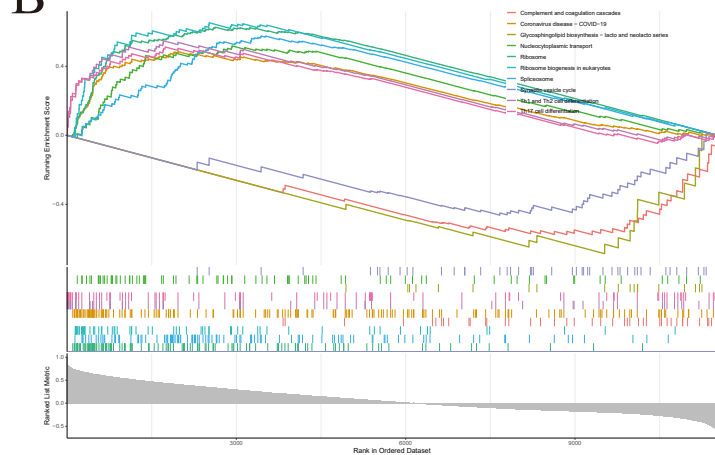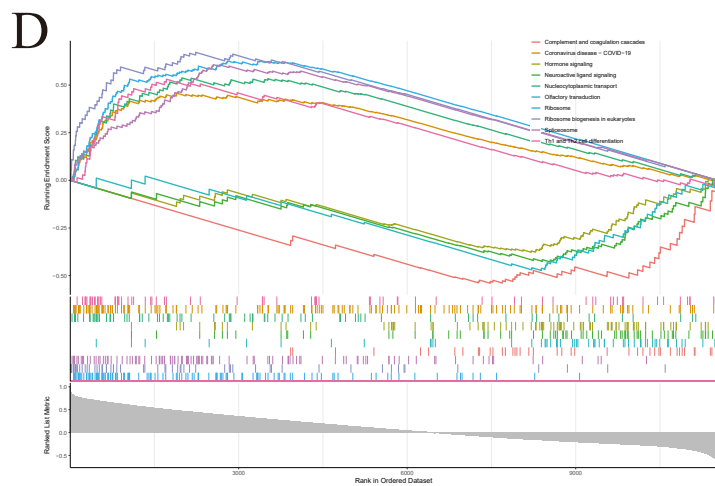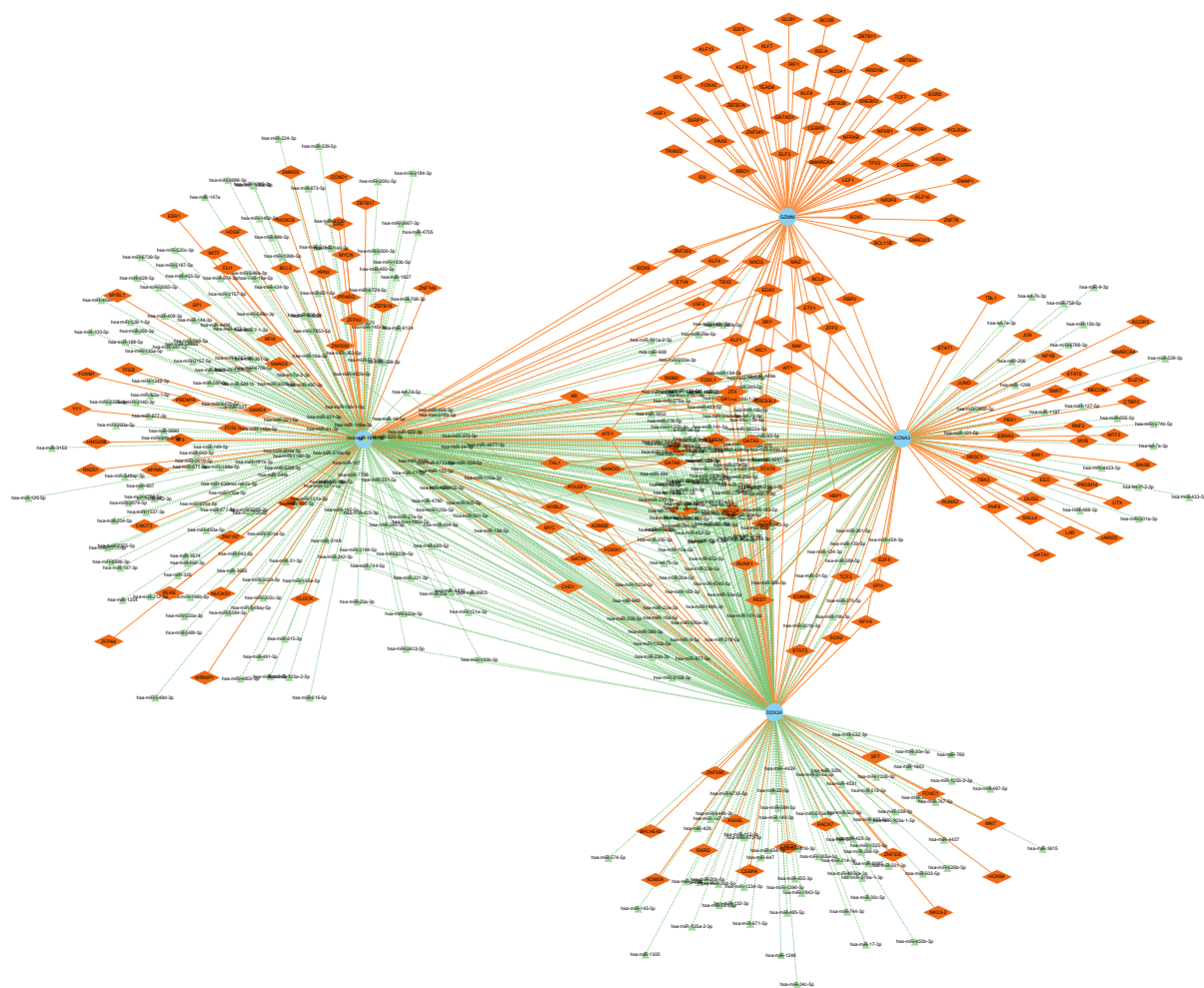

Supplement: Supplementary file 4 — Supporting Information 4 Figure S4: scRNA‐seq quality control (QC) and cell type annotation. [file HUMU-2026-4951633-s007.pdf]
